# Hundreds of conserved non-coding genomic regions are independently lost in mammals

**DOI:** 10.1093/nar/gks905

**Published:** 2012-10-05

**Authors:** Michael Hiller, Bruce T. Schaar, Gill Bejerano

**Affiliations:** ^1^Department of Developmental Biology and ^2^Department of Computer Science, Stanford University, Stanford, California 94305, USA

## Abstract

Conserved non-protein-coding DNA elements (CNEs) often encode *cis*-regulatory elements and are rarely lost during evolution. However, CNE losses that do occur can be associated with phenotypic changes, exemplified by pelvic spine loss in sticklebacks. Using a computational strategy to detect complete loss of CNEs in mammalian genomes while strictly controlling for artifacts, we find >600 CNEs that are independently lost in at least two mammalian lineages, including a spinal cord enhancer near *GDF11*. We observed several genomic regions where multiple independent CNE loss events happened; the most extreme is the *DIAPH2* locus. We show that CNE losses often involve deletions and that CNE loss frequencies are non-uniform. Similar to less pleiotropic enhancers, we find that independently lost CNEs are shorter, slightly less constrained and evolutionarily younger than CNEs without detected losses. This suggests that independently lost CNEs are less pleiotropic and that pleiotropic constraints contribute to non-uniform CNE loss frequencies. We also detected 35 CNEs that are independently lost in the human lineage and in other mammals. Our study uncovers an interesting aspect of the evolution of functional DNA in mammalian genomes. Experiments are necessary to test if these independently lost CNEs are associated with parallel phenotype changes in mammals.

## INTRODUCTION

The comparison of sequenced mammalian genomes revealed hundreds of thousands of conserved DNA regions that evolve under purifying selection, yet do not code for proteins (1–4). Many of these so-called conserved non-coding elements (CNEs) have *cis*-regulatory function by enhancing transcription in specific tissues or cell lines and time points (5–8). More and more regulatory CNEs are also implicated in human disease (9–11). CNEs contribute to organism fitness, as they are rarely lost during mammalian evolution (3,12). For example, the loss of ultraconserved elements (13) during rodent evolution has been estimated as 300-fold less likely than the loss of neutral DNA over a span of at least 60 million years (My) (12).

However, CNEs losses that do occur in evolution can be associated with phenotypic changes. For example, a CNE containing a transcriptional enhancer regulating the pleiotropic *Pitx1* gene is lost in independent stickleback populations and this CNE loss lead to the loss of pelvic spines in these populations (14). The loss of a conserved enhancer for the androgen receptor gene in humans is associated with the loss of sensory vibrissae and penile spines in humans (15) and the loss of a conserved forebrain enhancer in humans near the tumor suppressor gene *GADD45G* is correlated with the expansion of the human neocortex (15). Further examples of changes in *cis*-regulatory elements in *Drosophila* species underlie the independent gain and loss of wing pigmentation patterns (16) and the loss of larval trichomes (17). Both changes in coding and non-coding regulatory regions contribute to phenotypic evolution (18). Developmental genes in particular are often highly pleiotropic and mutations in such a gene would affect its function in many tissues of expression. In contrast, many *cis*-regulatory elements regulating developmental genes have no or little pleiotropy (about 50% of tested enhancers, see Supplementary Table S1). Mutations in such elements would change gene expression in only one tissue, leaving the function of a pleiotropic gene unaffected in other contexts (19–21), which potentially explains their higher frequency (∼80%) in loci underlying stickleback adaptations (22).

To systematically explore CNE loss during mammalian evolution, we developed an approach to detect ancestral functional elements that are conserved in many mammals, but are completely lost in other mammals. By obtaining a high-quality genome-wide list of CNE loss events, we discovered hundreds of CNEs that are independently lost in mammals, including losses in the human lineage. We show that CNE loss frequencies are not uniform and explore the characteristics of independently lost CNEs, which suggest a lower degree of pleiotropy.

## MATERIALS AND METHODS

### Alignments and CNEs

Our analysis using human as the reference species is based on the human (NCBI36/hg18 assembly) 44-way genome alignment, downloaded from the UCSC genome browser (23) (http://genome.ucsc.edu/). For the analysis where mouse is the reference species, we extended the mouse (NCBI37/mm9 assembly) 30-way genome alignment provided by the UCSC genome browser by the following assemblies to obtain an alignment with the same species as the human 44-way alignment except lamprey: sloth (choHof1), kangaroo rat (dipOrd1), gorilla (gorGor1), mouse lemur (micMur1), microbat (myoLuc1), pika (ochPri2), rock hyrax (proCap1), megabat (pteVam1), squirrel (speTri1), zebra finch (taeGut1), tarsier (tarSyr1), dolphin (turTru1) and alpaca (vicPac1). We updated the following species using newer assemblies: armadillo (dasNov2), cow (bosTau4), elephant (loxAfr2), guinea pig (cavPor3), horse (equCab2), human (hg19), medaka (oryLat2), opossum (monDom5) and rat (rn5). To build this alignment, we first obtained pairwise genome alignments using lastz (24) followed by chaining and netting (25) and then used multiz (26) with reciprocal-best nets for all species.

To obtain conserved regions, we used PhastCons (2) most-conserved elements in addition to regions that clearly align to outgroup species, keeping only elements that are ≥70 bp long. Because our focus is on non-coding conserved elements, we excluded all conserved elements that overlap exons contained in the UCSC tracks knownGene, refGene, mgcGenes, ccdsGene, ensGene, exoniphy and vegaGene. To further ensure that these elements are non-coding, we ran Blastx against the non-redundant protein (nr) database and discarded all elements having hits with an *E*-value of <0.01. We also discarded elements with overlap to snoRNAs, miRNAs, pseudogenes or transposons and portions of elements that are within 100 bp of exon flanks to avoid conserved splicing regulatory regions. As regions with close paralogy elsewhere in the reference genome are prone to mis-alignments of orthologous sequences, we further discarded elements that have a second BLAT hit with a score of ≥40. We always excluded regions on the mitochondrial chromosome as well as random and haplotype chromosomes.

To distinguish subsequent losses of an ancient element from the recent emergence of a conserved element in a clade, we discarded all elements that do not align to at least one of the following outgroup species: opossum (monDom5), platypus (ornAna1), chicken (galGal3), lizard (anoCar1) or zebra finch (taeGut1). The resulting human set comprised 231 653 CNEs totaling 55.7 Mb or 1.8% of the genome. The mouse set comprised 178 775 elements totaling 44.2 Mb or 1.6% of the genome.

To obtain a set of highly conserved elements, we kept only elements for which the fraction of rejected substitutions computed by GERP (27) is >50% and the conservation *P*-value from phyloP (28) is <10^−^^20^. These strict requirements are fulfilled for the top 37% of the human conserved elements. This set comprised 86 105 elements totaling 28.7 Mb or 0.9% of the human genome.

### Computational detection of CNE losses

Our approach is based on the multiple genome alignment. To detect complete CNE losses we searched for cases where the entire CNE has no alignment in a species. We required that each CNE lost in a particular species has aligning flanks upstream and downstream to that species. To exclude artifacts like assembly gaps that can mimic loss of a CNE we further excluded all cases where a region between the aligning flanks contains an assembly gap in the respective species. For species where we do not search for CNE losses, we interpret the absence of aligning CNE sequence as missing data in that species.

**Figure 1.**
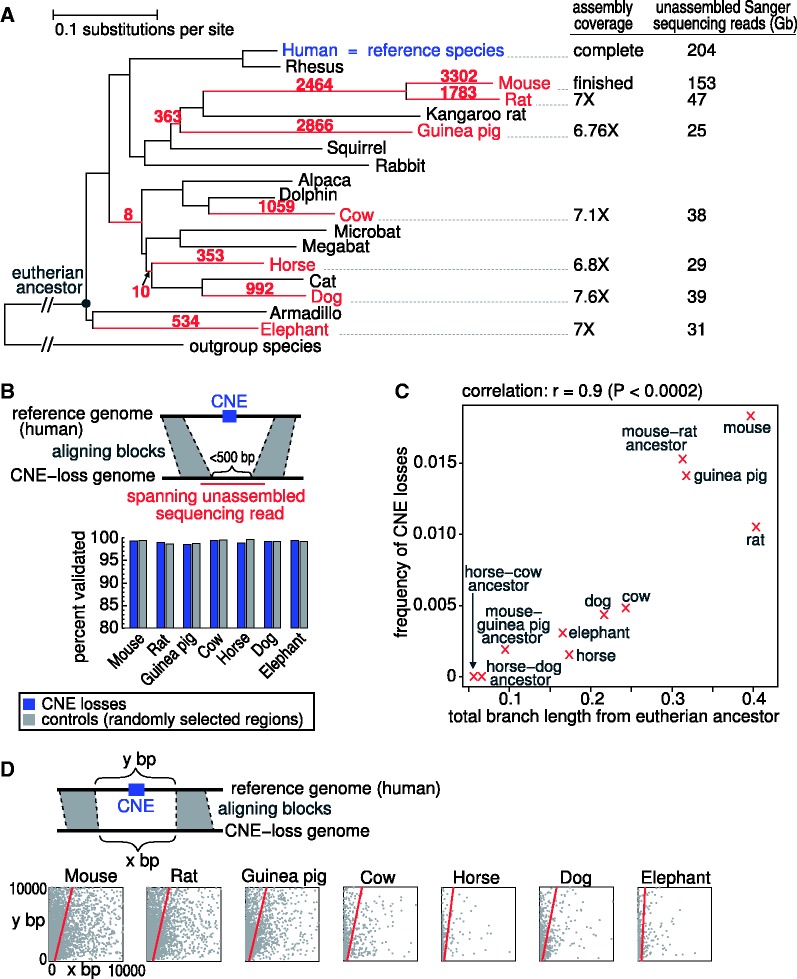
CNE losses in seven mammals. (**A**) For each CNE loss, we inferred the branch in the phylogenetic tree along which the loss likely happened by parsimony. The total number of observed losses is shown above each branch. Losses in branches leading to internal tree nodes have a loss or missing data for all descendant species. On the right, we show assembly coverage and available Sanger sequencing reads for the species where we search for CNE losses. (**B**) The vast majority of shorter assembly regions that comprise a CNE loss (region between the upstream/downstream aligning blocks is <500 bp) can be validated by unassembled sequencing reads that span the assembly region of CNE-loss species. (**C**) The frequency of CNE losses is strongly correlated with the branch length (neutral substitutions per site) from the eutherian (placental mammal) ancestor. (**D**) Plotting the distance between the aligning blocks in the reference (human genome, *y*-axis) and the CNE-loss (*x*-axis) genome shows that many CNE losses involve a large deletion. This trend is strongest in the species with the shortest branch length (horse, elephant). Linear regression line is in red.

We further discarded losses that have sequence similarity to any locus in the genome or to any of the unassembled sequencing reads (traces) of the respective species using lastz (24). This additional filter step excludes false losses that are due to genome assembly errors, errors in the multiple alignment and CNE translocations to a different locus. It should be noted that all of the species where we search for CNE losses have at least 25 GB of trace data (Figure 1A, Supplementary Table S7).

### Validating assembly correctness

We downloaded all unassembled Sanger sequencing reads from the NCBI trace archive for mouse, rat, guinea pig, dog, cow, horse and elephant. We used Blat (29) to map traces back to the respective genome requiring that traces align with at least 90% identity and that the alignment length is not more than 105% of the trace length. If a trace has multiple hits, we picked the best hit.

CNE losses that have a short distance between the aligning flanks in the CNE-loss genome were validated by searching for individual unassembled traces that span the entire region between the aligning flanks as well as 50 bp on either side in the CNE-loss genome. As the average size of traces is around 800 bp, we restricted this analysis to CNE losses where the region between the aligning flanks is <500 bp. Of all CNE losses, 32.4, 35.5, 44.2, 49.1, 68.7, 49.5 and 69.5% have aligning flanks separated by <500 bp for mouse, rat, guinea pig, cow, horse, dog and elephant, respectively. To check how the validation rates obtained for these CNE-loss regions compare to the rest of the genome, we randomly selected five control regions for each CNE loss. The size of these control regions equals the size of the CNE-loss region (distance between the aligning flanks). The control regions were picked from the same chromosomes/scaffolds that have CNE losses and were selected not to overlap assembly gaps.

### Simulating neutral evolution without large insertions or deletions

Simulating genome evolution was done using Evolver (http://www.drive5.com/evolver/) with the parameters given in http://www.drive5.com/evolver/evotoy.tar.gz. We used human chromosome 1 as our ancestral genome with all coding RefSeq genes. Functional non-coding regions were placed in the ancestral genome using Evolver, so that 5% of the genome is covered with regions that evolve under purifying selection. To evolve the ancestral genome, we used this topology ((human:0.14, mouse:0.35):0.02, horse:0.15), where the branch lengths correspond to the real mammalian phylogeny (Supplementary Figure S1). We selected mouse and horse because these two species have the longest and shortest branch length, respectively, among the species where we search for CNE losses, yielding an upper and lower bound.

We randomly picked 1000 non-coding conserved regions. Along the human lineage, those 1000 regions evolved under constraint and all events (substitutions, insertions/deletions of any size as well as transposon insertions) were allowed to occur. Along the mouse and horse lineage, those 1000 regions evolved neutrally allowing only substitutions and insertions/deletions of 3 bp or less to occur. To do that, we set all rates for insertions/deletion larger than 3 bp to 0 and we turned off transposon insertion events. After evolving the human genome, we extracted the human sequence of these 1000 regions and used lastz to search for matches in the evolved mouse and horse genome. We counted how many of the 1000 human regions have a lastz match and reported the average of five independent iterations of the entire evolutionary process.

### Inferring the loss branches and computing the number of independent losses

We used parsimony to infer along which branch in the phylogenetic tree a CNE was lost. For each CNE, we removed species with missing data from the tree. A loss in sister species in the pruned phylogenetic tree (such as a loss in mouse and rat) is then inferred to be a single loss in the most recent common ancestor of these species (mouse–rat ancestor). To obtain the number of independent loss events, we counted how many different branches in the tree are labeled with a loss. In addition we assured for each independent loss that there is always at least one species that is situated between the different loss branches that conserves the CNE. For example, we only considered a CNE to be lost independently in the mouse and guinea pig lineage if the CNE is conserved in at least one of rat or kangaroo rat.

### Expected number of independent CNE losses using uniform loss frequencies

We calculated the exact number of expected independent CNE losses. Let *L*_A_ be the number of CNE losses in the branch leading to the phylogenetic tree node A. Then *f*_A_ = *L*_A_/*N*_A_ is the frequency of CNE losses in this branch where *N*_A_ is the total number of CNEs with the potential to have a loss in A. *N*_A_ is usually smaller than the total number of CNEs due to missing data which prevents some CNEs from having this loss (e.g. a CNE with missing data for mouse or rat, by definition, cannot have a loss in the mouse–rat ancestor). Given the frequency of losses in two independent branches A and B, we compute the expected number of CNEs independently lost in these branches as *f*_A_ × *f*_B_ × *N*_AB_, which explicitly tests the assumption that CNE losses happen independently. *N*_AB_ is the number of CNEs with the potential to have an independent loss in A and B. *N*_AB_ is usually smaller than the minimum of *N*_A_ and *N*_B_ due to missing data and the fact that an independent loss requires CNE conservation for at least one species between the loss branches (e.g. an independent CNE loss in the mouse–rat ancestor and in the guinea pig lineage requires CNE conservation in kangaroo rat). To obtain the total expected number of two independent losses, we summed over all possible independent lineage combinations. The expected number of three independent losses was computed analogously.

### CNE loss simulation

We simulated CNE losses along the mammalian phylogeny using the real number of losses in each lineage. Each iteration of the simulation starts with a list of triplets (CNE, species, label) for each CNE and all species excluding those with missing data. All labels are initially set to ‘conserved’. Then we sort all observed loss events by depth of the node in which the loss branch ends in ascending order (root has depth = 0). This sorted list reflects the relative evolutionary order of the CNE losses. For example, a loss in the common ancestor of mouse and guinea pig happened before a loss in the mouse–rat ancestor, which in turn happened before a loss in the mouse lineage. Then, for each loss event in the sorted list, we assigned it to a randomly picked ‘valid’ CNE and set the labels for all species included in the loss event from ‘conserved’ to ‘lost’. If the assignment is invalid, we randomly pick another CNE until a valid assignment is found. The assignment can be invalid for the following three reasons. First, this CNE might already have a ‘lost’ label for one of the species included in the given loss event (e.g. as mouse–rat losses are assigned before single mouse losses, the CNE might already be labeled with a mouse loss). Second, the loss event might be in conflict with missing data for certain species for this CNE. For example, a loss in the mouse lineage cannot be assigned to a CNE with missing data for mouse. Likewise, a loss in the mouse–rat ancestor cannot be assigned to a CNE with missing data for mouse or rat. Third, assigning the loss event to this CNE can lead to inferring a different loss branch by parsimony. For example, a CNE that is currently labeled with a loss in the mouse lineage cannot be labeled with another loss in the rat lineage as parsimony would infer a single loss in the mouse–rat ancestor. After all the loss events are assigned to randomly picked CNEs, we use parsimony to infer the loss branches. By design this simulation yields in each iteration exactly the same loss branches that comprised the input, thus the total number and identity of loss events remain unchanged. Then, we count how many independent losses happened (in total and for each combination of lineages). An independent loss arises by randomly assigning two or more different losses to the same CNE. We performed 10 000 iterations to obtain a distribution for the number of independent losses. We further tested the simulation using a reverse relative evolutionary order of the CNE losses as well as randomly picking CNE losses from the list in each iteration and obtained highly similar distributions (not shown).

### Association of CNE loss with gene loss

We assigned each independently lost CNE to the nearest upstream or downstream transcription start site of a coding gene included in the Ensembl database (30) as enhancers regulate genes in an orientation-independent manner (11). We downloaded orthologous genes for mouse, rat, guinea pig, cow, horse, dog and elephant from Ensembl. Then we asked for the CNE-loss species if the gene assigned to the lost CNE has an ortholog. We manually inspected the cases where the nearest gene lacked an orthology assignment to the CNE-loss species and excluded cases where the gene presence is indicated by a clear protein-to-genome alignment using Blat or where the gene is absent due to assembly gaps. For the cases represented in Supplementary Table S5, we found mutations that clearly inactivate the gene.

### Ancestry and strength of constraint

We inferred that a CNE predates the mammalian ancestor if it aligns with at least 80% of its length to any of the following species: chicken, zebra finch, lizard, frog, zebrafish, tetraodon, fugu, stickleback or medaka. To compute the strength of constraint we used GERP (27) and computed the fraction of rejected substitutions (number of rejected/number of expected substitutions). As deeper CNE ancestry is associated with less CNE losses and is positively correlated with constraint (31), we removed the effect of the confounding variable ‘ancestry’ by using only sequences of eutherian species as input to compute the strength of constraint. Furthermore, to exclude the confounding variable ‘length’ which also influences the constraint (31), we compared size-matched sets of CNEs by randomly picking for each independently lost CNE a CNE with no losses and a CNE with lineage-specific losses having the same size (this procedure gives exactly the same size distribution for all three sets).

### Pleiotropic enhancers

We downloaded the regions bound by the enhancer component p300 in developing (mouse at e11.5) forebrain, midbrain and limb tissue from the supplement of (32), used liftOver (minMatch = 0.25) to convert mouse to human (hg18) coordinates and excluded p300 regions overlapping exons to avoid pleiotropy with genes. A total of 2108 regions were considered to be pleiotropic enhancers since p300 regions from 2 or 3 tissues overlap by at least one base. In all, 6249 regions are bound by p300 in only one tissue and were considered as less pleiotropic enhancers. We use less pleiotropic instead of non-pleiotropic because these enhancers could also drive expression in a tissue that was not assayed. For length, we downloaded PhastCons (2) most-conserved vertebrate elements from the UCSC genome browser, merged those elements that are <15 bp apart and counted the sum of bases in these conserved elements overlapping individual p300 elements. For ancestry, we inferred that a region predates the mammalian ancestor if it aligns to chicken, zebra finch, lizard, frog, zebrafish, tetraodon, fugu, stickleback or medaka in the human 44-way alignment. To compute the strength of constraint on a p300 region, we applied GERP (27) to all PhastCons elements that overlap a region using only sequences of eutherian species as above.

### LacZ reporter assay

The human CNE downstream of *GDF11* was amplified using primers 5′-CACCAGATCTTTGGTGCCTCTTCAG-3′ and 5′-TCTCCTCTTTTGCACAATTGTTTCTCA-3′ and TOPO cloned into pENTR/D (Invitrogen). The insert was then cloned into a version of pHSP68-LacZ containing a Gateway cassette (reading frame A, Invitrogen) upstream of the HSP68 basal promoter, pHSP68-LacZDest. Transgenic mice were generated by pronuclear injections of FVB embryos (Xenogen Biosciences). Embryos were harvested at embryonic day 13.5 and whole-mount lacZ staining was performed as described in (33) except the embryos were cleared in graded 15% and 30% sucrose washes.

## RESULTS

### Computational approach to accurately detect and validate CNE losses

Using a multiple whole-genome alignment with human as the reference species, we search for the complete loss of a CNE, which we define as the absence of aligning sequence embedded in a syntenic alignment of the regions flanking this CNE. To obtain a high-quality set of bona fide CNE losses, we applied several filtering steps. First, as assembly artifacts can be mistaken for the absence of a CNE, we restrict ourselves to detecting losses in species having high-quality genome assemblies: mouse, rat, guinea pig, cow, horse, dog and elephant (Figure 1A and Supplementary Figure S1). These species have genomes with a high coverage of at least 6.7X. Second, we only considered CNE losses where the region between the aligning flanks contains no assembly gap in this CNE-loss genome. Third, to further exclude assembly or alignment errors as well as the translocation of the CNE to a different genomic locus, we used the wealth of unassembled Sanger sequencing reads (traces) that is deposited in the NCBI trace archive (at least 25 GB of sequence per species; Figure 1A). We required that a sensitive search against the entire genome as well as all unassembled sequencing reads detects no sequence similarity to the CNE sequence. This means that a lost CNE has no sequence similarity to the entirety of sequenced DNA for this species. We did not search for CNE losses in mammalian species other than the seven listed above because they often have low-coverage genomes. For these species, we consider alignment to the CNE as CNE presence, but we interpret the absence of a CNE (which can be due to artifacts) as missing data. To distinguish loss of an ancient CNE from more recent CNE gain, we only analysed CNEs that are conserved in outgroup species (opossum, platypus, chicken, lizard or zebra finch). Using this strategy, we analysed a total of 231 653 CNEs and found 13 085 CNEs (5.6% of 231 653) that are lost in at least one of mouse, rat, guinea pig, cow, horse, dog and elephant (Figure 1A).

### Validating assembly correctness using unassembled traces

To further provide evidence that these losses are not artifacts, we validated the assembly regions that comprise the lost CNE by using the unassembled Sanger sequencing reads. After mapping all sequencing reads back to the respective genome, we expect to find single unassembled reads that span the entire CNE-loss region if that region is correctly assembled and is smaller than the length of such reads. Indeed, we found in total for 99.02% of such regions single spanning reads (Figure 1B). Furthermore, randomly selected regions of the same size showed highly similar validation rates, indicating that these CNE-loss regions are representative for the whole genome and not enriched for problematic assembly regions. The validation rate is also highly similar between species (between 98.5% and 99.5%), indicating that assembly correctness for these CNE-loss regions does not differ between the 7 species. This indicates that our stringent parameters yielded a high-quality set of bona fide CNE losses.

### CNE loss frequency is correlated with the branch length

We used the parsimony principle to infer the branch in the phylogenetic tree along which the 13 085 CNE losses likely happened (Figure 1A). Parsimony assumes that a common loss in two sister species (such as mouse and rat) happened in the ancestor of both species. Consistent with previous findings (3,12), the CNE loss frequency is around 1% (Figure 1C). We observe a significant correlation between the CNE loss frequency and the branch length from the eutherian ancestor measured in neutral substitutions per site (*r* = 0.9, *t*-statistic = 6.1, df = 9, *P* < 0.0002; Figure 1C).

### CNE losses often involve deletions

The CNE losses in mouse, rat, guinea pig, cow, horse, dog and elephant could have happened during a long evolutionary time span (e.g. a loss in horse could have happened during the last ∼83 My since the horse lineage split from the dog/cat lineage, Supplementary Figure S1). The molecular mechanism leading to complete CNE loss (no detection of aligning sequence) can involve (i) large deletions that remove the entire or large parts of the CNE at once; or (ii) the accumulation of many small events (base substitutions, small insertions/deletions) that change the CNE sequence to an extent that alignment methods can no longer detect any sequence similarity. To address which type of events is mainly responsible for CNE loss, we compared the distance between the aligning flanks in the human and the CNE-loss genome. If the accumulation of many small events were mainly the underlying cause of our detected CNE losses, we would expect that both distances are roughly the same. In contrast, we observed a strong and consistent trend that the distance in the CNE-loss genome is substantially shorter in all of the seven species (Figure 1D). The shorter distance in the CNE-loss genomes indicates that larger deletions are often involved in complete CNE loss.

To further test this, we simulated genome evolution to ask if the accumulation of substitutions and small insertions/deletions alone would be sufficient to change the CNE sequence to an extent that no sequence similarity to the human CNE sequence can be detected anymore (see Materials and Methods section). This simulation shows that only 0.2% of the CNEs for a short branch length (such as horse) and 18.9% of the CNEs for a longer branch length (such as mouse) would have no detectable sequence similarity (complete loss in our pipeline) (Supplementary Figure S2). This agrees with the above observation that complete CNE losses mostly involve large deletions, especially for species with short branch lengths.

### Hundreds of independent CNE losses

**Figure 2.**
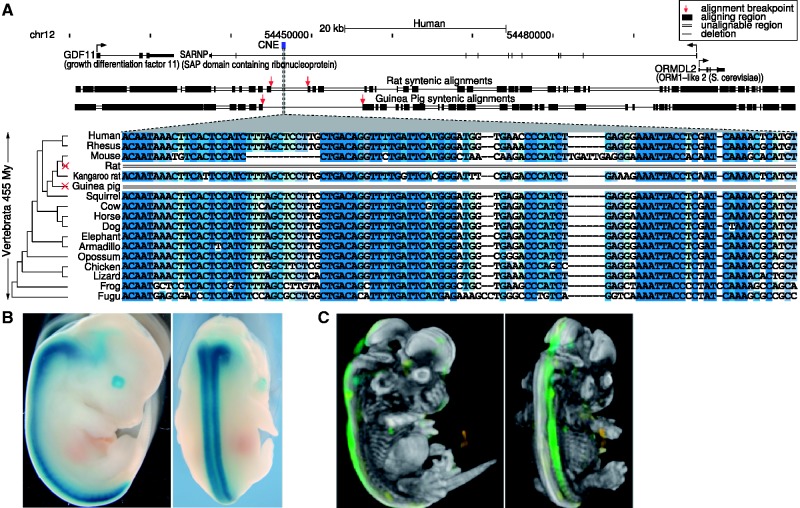
An independently lost CNE is a transcriptional enhancer in development. (**A**) The CNE is lost independently in the rat and guinea pig lineage (lacking any sequence similarity to the genome and all unassembled traces) but is conserved over ∼450 My of vertebrate evolution. The top part shows the location of the CNE (blue) in the human genome together with the exon–intron structures of the surrounding genes (arrows indicate the transcription start site). Below is a graphical representation of pairwise genome alignments to rat and guinea pig showing that the genes but not the CNE align to both species. Syntenic aligning regions in rat and guinea pig are shown as black boxes, a single line indicates a deletion between aligning blocks and a double line indicates that the region contains sequence that does not align. Red arrows mark the ends of the up- and downstream aligning blocks and suggest that independent events led to the CNE loss in rat and guinea pig. The bottom part shows the sequence alignment where darker blue shades indicate higher sequence identity in an alignment column. (**B**) A transcriptional enhancer assay in transgenic mouse embryos reveals *lacZ* reporter expression, showing that the CNE is a spinal cord enhancer at embryonic day 13.5 (7 out of 7 embryos). (**C**) The expression pattern in (B) is consistent with *in situ* hybridization data of *GDF11* at embryonic day 13.5, suggesting that this CNE is a regulatory element for *GDF11*. Data from Image Series 100047449, Allen Developing Mouse Brain Atlas, Seattle (WA): Allen Institute for Brain Science. ©2009. http://developingmouse.brain-map.org.

Our screen uncovered CNEs that are independently lost in mammalian lineages (Figure 2A, Supplementary Figure S3). Of the 13 085 CNEs, we found a total of 590 CNEs lost twice independently, 28 CNEs lost 3-times independently and 1 CNE that is lost in 4 independent lineages (Supplementary Figure S3C). These 619 CNEs are found on all chromosomes except Y, which is not sequenced in most mammals (Supplementary Figure S4). The validation rate for the assembly regions comprising CNEs with independent losses is highly similar to the assembly regions comprising lineage-specific CNE losses (Supplementary Figure S5), suggesting that independent CNE losses are not associated with problematic assembly regions.

### Loss of a transcriptional enhancer near *GDF11*

To examine the elements for function, we selected a CNE retained in both human and mouse. This CNE is independently lost in rat and guinea pig and is ∼22 kb downstream of the transcription start site of the developmental gene *GDF11* (growth differentiation factor 11) (Figure 2A). *GDF11* is involved in skeletal patterning (34) and influences Hox gene expression to determine neuronal identity and differentiation in the spinal cord (35,36). In fish, synteny between this CNE and *GDF11* is conserved, while synteny to the downstream genes is broken (Supplementary Figure S6). We used a transgenic enhancer assay in mouse to test for *cis*-regulatory function. We found that this CNE is a spinal cord enhancer at embryonic day 13.5 (7 of 7 transgenic embryos) with an expression pattern very similar to that of *GDF11* at the same developmental time point (Figure 2B and C). The loss of this enhancer in rat and guinea pig may have caused changes in the *GDF11* expression pattern or expression level.

### Genomic regions with multiple independent CNE loss events

**Figure 3.**
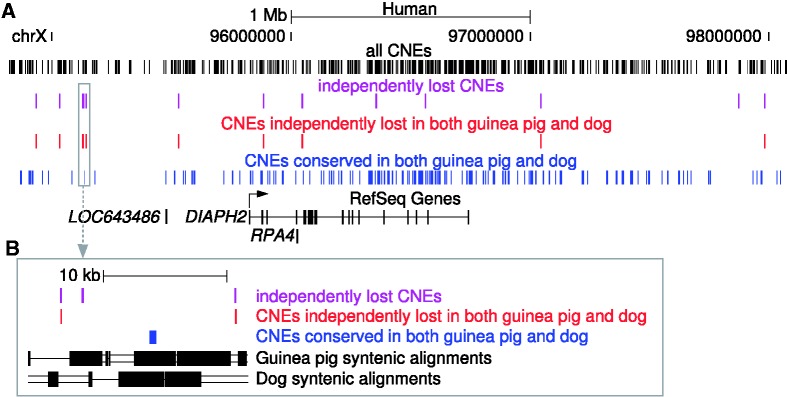
Many independent CNE losses around the *DIAPH2* gene in guinea pig and dog. (**A**) The *DIAPH2* locus contains the largest concentration of independent CNE loss events with 13 losses within 5 Mb. Note that each pair of CNEs that are independently lost in guinea pig and dog (red) is separated by at least one CNE that is conserved in guinea pig and dog (blue), which shows that all nine CNEs are lost by independent events (as opposed to being lost by a small number of large loss events that remove several CNEs at once). None of these 13 CNEs show any evidence for transcription. *DIAPH2* is likely a functional gene in both guinea pig and dog. Other genes in this locus are the non-coding RNA gene LOC643486 and the coding gene *RPA4* (replication protein A4, 30 kDa). *RPA4* is likely a primate-specific gene, while the CNEs all have at least placental mammal ancestry. (**B**) Zoom-in of the grey-boxed region in (A) illustrates how the presence of a CNE conserved in guinea pig and dog (blue) shows that the two CNEs independently lost in guinea pig and dog (red) are lost by two separate events in guinea pig and dog. The representation of pairwise alignments is as in Figure 2A.

We investigated the clustering of independently lost CNEs by sliding a window over the genome and counting the number of such CNEs in each window (Supplementary Table S2). A 5 Mb window around *DIAPH2* (diaphanous homolog 2), a gene linked to premature ovary failure in human (37), contains a striking 13 independently lost CNEs, which is the highest concentration genome wide (Figure 3). Interestingly, 9 of these 13 CNEs are lost in both guinea pig and dog (lacking any match both in the genome and in all unassembled trace reads, as described above). We found that for each adjacent pair of these nine CNEs, at least one CNE with clear conservation in both guinea pig and dog is located in-between. This shows that nine independent loss events in guinea pig and dog led to these nine independent CNE losses and excludes the possibility that a few large events deleted many of these nine CNEs at once. Noteworthy, *DIAPH2* has an intact exon–intron structure and is likely a functional gene in both guinea pig and dog, indicating that these nine CNE losses are not associated with loss of *DIAPH2*.

**Figure 4.**
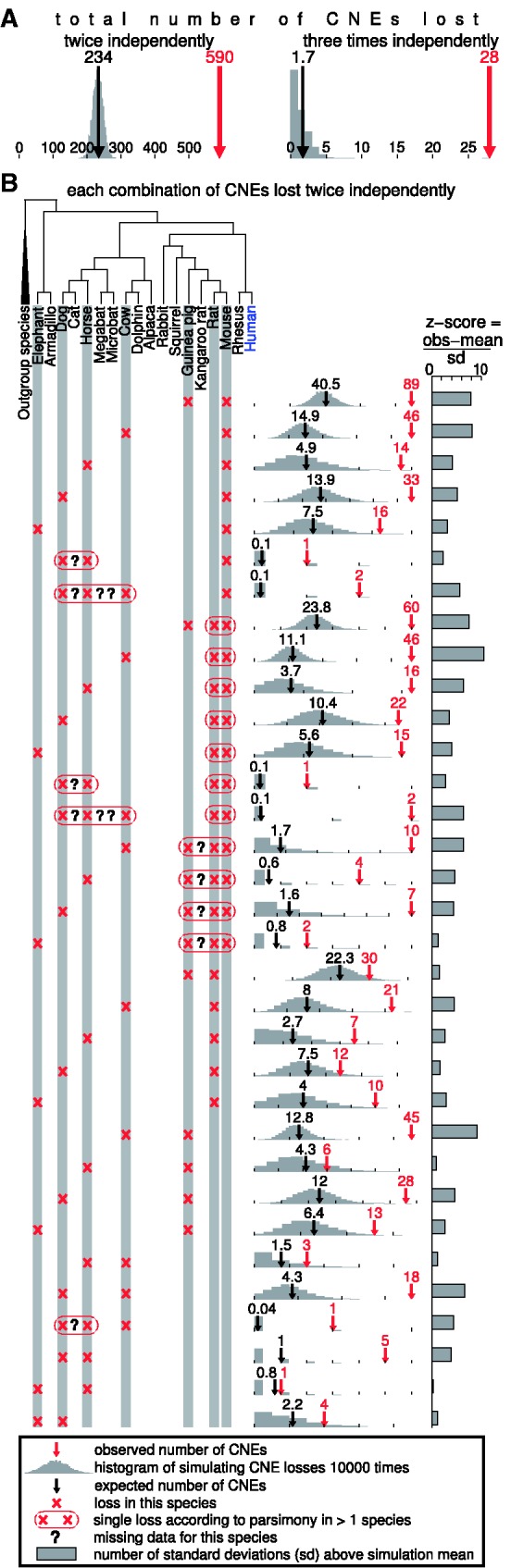
CNE loss frequencies are not uniform. (**A**) The observed number of CNEs lost twice and three times (red arrow) is significantly more than expected from two methods using a uniform loss model (black arrow is the calculated point estimate, histogram of simulations in grey). The observed number of 590 CNEs lost twice corresponds to a *z*-score (number of standard deviations above the simulation average) of 24 and the observed number of 28 CNEs lost 3-times corresponds to a *z*-score of 20. The maximum number of CNEs lost twice and 3-times in the simulation is 285 and 9, which gives an empirical *P*-value <0.0001 for the observed independent CNE losses. (**B**) All observed combinations of two independent CNE losses. The rightmost chart boundary is either the maximum of the 10 000 simulation iterations or the observed number of losses. Outgroup species are opossum, platypus, chicken, lizard or zebra finch. Human was used as the reference species.

We also tested if the sets of CNEs that are independently lost in specific lineage combinations (e.g. mouse and cow, see Figure 4B) are linked to genes enriched in specific functions using GREAT (38). We did not obtain significant enrichments for any set, which might be partially due to the lack of power caused by the small set sizes.

### CNE loss frequencies are not uniform

Given that 13 085 CNEs are lost in at least one species, we asked how many independent CNE losses could be expected under a uniform loss model. To address this, we both calculated a point estimate and obtained the expected number by simulating CNE losses (see Materials and Methods section); both methods yield highly similar results (Figure 4 and Supplementary Table S3). We found that under these uniform CNE loss frequencies only ∼234 CNEs lost twice and ∼2 CNEs lost 3-times are expected, which is significantly less than the observed 590 and 28 CNEs with 2 and 3 independent losses (empirical *P*-value < 0.0001, Figure 4, Supplementary Figure S7). This large difference between observed and expected independent losses cannot be explained by overlapping large-scale deletions in independent lineages that commonly remove numerous CNEs (Supplementary Table S3). To further exclude that this observation is caused by the slowly evolving tail of neutral DNA, we re-analysed a subset of CNEs under extreme sequence conservation. In this extremely conserved subset, we observed 62 CNEs lost twice and 1 CNE lost 3-times, which is significantly more than the ∼15 CNEs with 2 and 0.04 CNEs with 3 losses expected under uniform loss frequencies (*P* ≤ 0.04; Supplementary Figure S8, Supplementary Table S4). These observations clearly indicate that certain CNEs have a higher chance to be lost than others.

### Independent CNE losses are not associated with nearby gene losses

Why are certain CNEs more likely to be lost? One possibility to explain the non-uniform CNE loss frequency is that these CNEs contain regulatory elements for a gene and if this gene is lost these regulatory elements would be rendered dispensable. To address this, we assigned each of the 619 independently lost CNEs to the gene with the nearest transcription start site and asked how many of these genes are lost in the species lacking the CNE. We found only 11 CNEs (1.8% of 619) where the nearest gene is inactivated in at least 1 of the CNE-loss species (Supplementary Table S5). Furthermore, for only 5 of these 11 CNEs, the nearest gene is lost in all species lacking the CNE. This suggests that nearby gene loss explains only a small fraction of the independent CNE losses.

### Length, constraint and ancestry of independently lost CNEs indicates less pleiotropy

**Figure 5.**
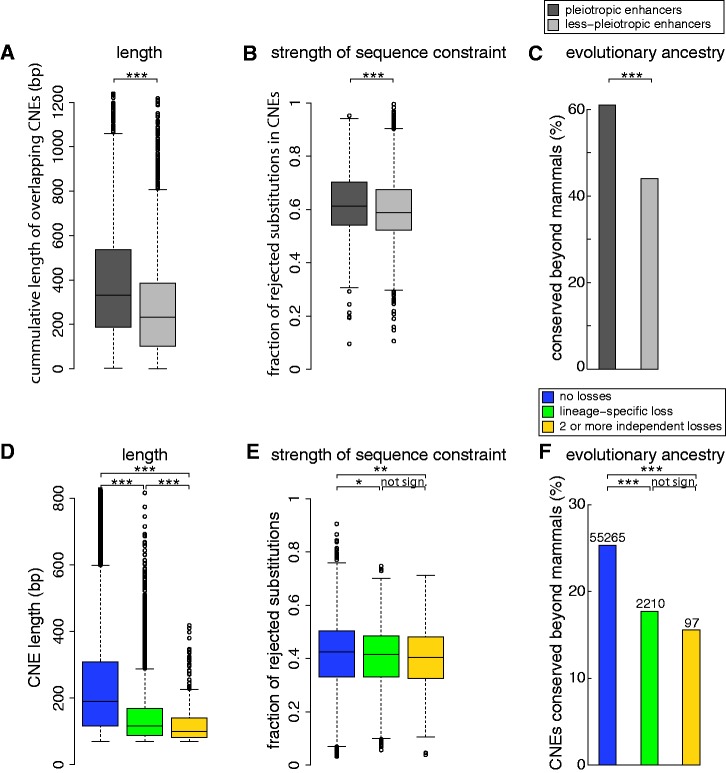
Differences in features indicative of pleiotropy between CNEs with and without losses. (**A–C**) We compared less pleiotropic enhancers (regions bound by p300 in only one of developing forebrain, midbrain or limb tissue (39)) to pleiotropic enhancers (bound by p300 in two or three of these tissues). (A) Less pleiotropic enhancers have fewer bases overlapping CNEs. (B) Less pleiotropic enhancers overlap CNEs that have a lower level of constraint, as measured by the fraction of substitutions that is rejected by purifying selection (calculated with GERP (27)). They are also less often extremely constrained (for 19.8% versus 25.7% more than 70% of the substitutions are rejected by selection). (C) Less pleiotropic enhancers align less frequently to non-mammalian vertebrates (chicken, zebra finch, lizard, frog or fish), showing that they are evolutionarily younger. (**D–F**) We compared CNEs with no detected losses to CNEs with lineage-specific and independent losses. The CNEs with losses show signatures indicative of less pleiotropy. (D) CNE length decreases with the number of loss events. (E) CNEs with loss events are depleted in extremely constrained elements. (F) CNEs with loss events are evolutionarily younger. Box plots visualize the distribution in (A), (B), (D) and (E). Bar charts are shown in (C) and (F). For visualization clarity the *Y*-axis is cut at a size of 1200 bp in (A) and 800 bp in (D). Wilcoxon rank-sum test was used in (A), (B), (D), (E), chi-square test in (C) and (F). ***P-value < 0.0001; **P-value < 0.01; *P-value < 0.05.

Pleiotropy of a *cis*-regulatory element, which we define here as driving expression in more than one anatomical structure, can be a reason for preserving a regulatory element, even when some of its functions become dispensable. We used genomic regions bound by the enhancer protein p300 in mouse forebrain, midbrain and limb tissue at embryonic day 11.5 (39) to obtain a set of 2108 pleiotropic (regions bound by p300 in 2 or 3 tissues) and 6249 less pleiotropic (bound by p300 in only 1 of the 3 tissues) enhancers and explored their characteristics. We compared length, strength of constraint and evolutionary ancestry and found that less pleiotropic enhancers overlap shorter CNEs, have a lower level of sequence constraint and are evolutionary younger (Figure 5A–C).

Next, we compared these characteristics between CNEs with no detected losses, lineage-specific losses and independent losses (Figure 5D–F). First, we found that CNEs with losses are shorter (average length 246, 141 and 121 bp for CNEs with no detected, lineage specific and independent losses, respectively). We cannot completely exclude the possibility that long CNEs are less likely completely lost as this may require a large deletion. However, the median deletion lengths (estimated by distance difference between the aligning flanks in the human and the CNE-loss genome) of 2870 bp (average 6355 bp) exceeds the median CNE length of 181 bp (average 238 bp, only 7 of 236 165 CNEs are longer than 2870 bp) 15-fold, suggesting that most deletions are much longer than the CNEs. Second, CNEs with losses have a slightly lower level of constraint (average fraction of rejected substitutions: 0.415, 0.405, 0.4) and contain fewer elements that evolve under extreme sequence constraint (percent CNEs where >60% of all substitutions are rejected: 7.1, 4.4, 2.4%). Third, CNEs with losses are evolutionary younger (25.3, 17.7 and 15.5% align to non-mammalian vertebrates, indicating that they predate the mammalian ancestor).

In summary, CNEs with lineage specific and in particular independent losses show characteristics similar to less pleiotropic enhancers. In contrast to length, sequence constraint and ancestry, differences in GC content or dinucleotide frequencies are marginal between CNEs with no losses, lineage specific or independent losses (Supplementary Figures S9 and S10).

### Independent CNE losses involving the human lineage and within primates

**Figure 6.**
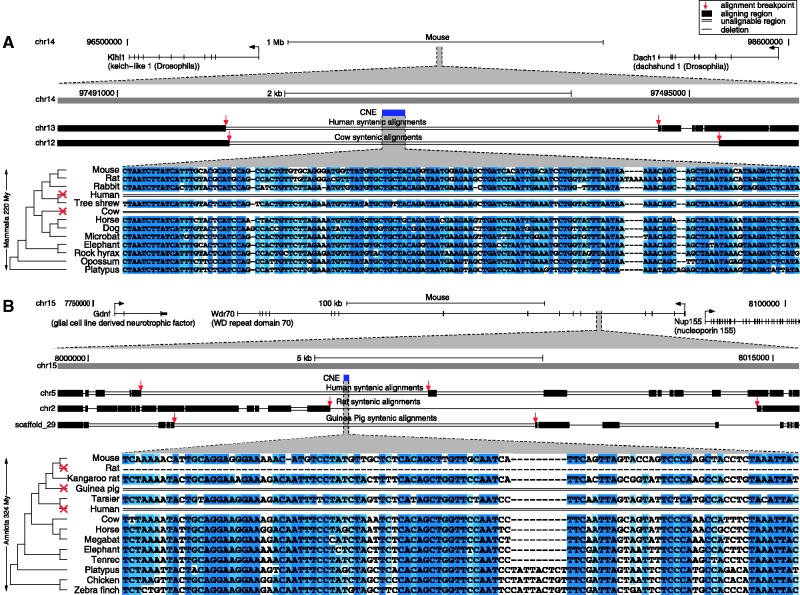
Examples of CNEs lost in the human and other independent lineages. (**A**) Two independent losses in the human and cow lineage. (**B**) Three independent losses in the rat, guinea pig and human lineage. The losses in the human lineage likely happened in the human—marmoset ancestor in both cases. Legend as in Figure 2A.

To detect complete CNE losses in the human lineage, we used a whole-genome alignment of the same set of species but having mouse as the reference species. Using a reference species other than human is necessary as human CNE losses cannot be detected in a human-referenced alignment (15). We found a total of 4584 (2.6% of 178 775) CNEs with at least 1 loss. Overall we observed 183 CNEs lost twice, 6 lost 3-times and 1 lost 4 times (Figure 6, Supplementary Figure S11 and Supplementary Table S6). This includes 35 CNEs that have a loss in the human genome and another independent loss in a different lineage.

The comparison of the human and Neandertal genome detected several genomic regions under positive selection in recent human evolution (40). Although our CNE losses are usually much older than these selective sweeps, we found that the human loss region of 2 of these 35 CNEs overlaps a selective sweep region (1 region overlapping the *EBHF1* and *OTX1* genes, 1 overlapping *WDR70* (Figure 6B); highlighted in Supplementary Table S9), which may indicate that these regions experienced recurrent changes along the human lineage.

Most of the mammalian species in our human and mouse referenced alignments diverged from each other over 60 My ago (Supplementary Figure S1A). To test if independent CNE losses also occurred in lineages that diverged more recently, we focused on the primate clade and restricted our search to CNE losses in only human, chimpanzee, orangutan, rhesus and marmoset. Those primates have high-quality genomes (coverage of at least 5.1X) and at least 25 GB of unassembled trace reads each (Supplementary Table S7). We found a total of 22 CNEs that are independently lost in 2 primate lineages, including 6 CNEs with losses in the human and another independent primate lineage (Supplementary Figure S12, Supplementary Table S8). This shows that several independent CNE losses also happened during more recent evolution in the primate clade.

Applying the point estimate and CNE loss simulation to these two sets of CNE losses, we consistently found that some CNEs have a significantly higher chance to be lost than others (all empirical *P* < 0.0001; Supplementary Figures S11 and S12). We also observed that CNEs with losses are (i) shorter (average length 251, 150 and 115 bp for CNEs with no detected, lineage specific and independent losses for the set using mouse as reference genome, respectively; 249, 145, 123 bp for the primate CNE loss set); (ii) less constrained and depleted in extremely constrained elements (percent CNEs where >60% of all substitutions are rejected: 11.9, 3.2, 1.6% for the set using mouse as reference genome; 11.3, 0, 0% for the primate CNE loss set); and (iii) evolutionarily younger (percent of CNEs aligning to non-mammalian vertebrates: 24.8, 16.3, 13.7% for the set using mouse as reference genome; 24.6, 14.7, 18.1% for the primate CNE loss set) (Supplementary Figure S13). This confirms the observations made above and supports generalizability of these trends.

## DISCUSSION

We present a computational strategy to detect CNE losses while excluding artifacts that can be mistaken for CNE loss. This strategy is not restricted to CNEs and can be extended to other functional elements as well as genomes of other clades. We find that most of these complete CNE losses involve large deletions in all species. We show that the sequenced mammalian genomes harbor hundreds of CNEs that are independently lost in a non-uniform fashion.

Why are certain CNEs more likely to be lost? Losing a conserved and thus functional element is expected to have deleterious effects, which should be selected against, as shown in (12). However, CNEs can be lost in lineages without deleterious effects if their function becomes dispensable. First, dispensability might arise by turnover of *cis*-regulatory elements, which involves the emergence of a functionally equivalent element at a new locus, creating redundancy between the new and the ancestral element, followed by the loss of the ancestral element. This situation was observed for enhancers of the *yellow* gene in Drosophila species (41). Second, CNE dispensability can also be due to the loss of a gene that is regulated by *cis*-regulatory elements overlapping these CNEs. However, we found that only a small fraction of the independently lost CNEs is associated with gene loss. Third, a CNE would become dispensable if it contains a *cis*-regulatory element for a tissue in which the expression of the functional target gene is not necessary anymore. Loss of such a CNE can lead to gene expression changes, which are thought to be a main factor for phenotypic evolution (19–21). It is conceivable that the independent dispensability of a CNE, which can lead to independent CNE loss, is associated with independent phenotype changes. It is interesting to note that there are a number of known repeated phenotype changes (16,21,42–44), providing another intriguing potential explanation for independent CNE losses.

Phenotypic changes would more likely lead to the dispensability and subsequent loss of CNEs with little or no pleiotropy as purifying selection would preserve a pleiotropic CNE even if one of its functions became dispensable. The comparison of pleiotropic to less pleiotropic enhancers showed that less pleiotropic enhancers are shorter, have a lower level of sequence constraint and are evolutionary younger. We observed the same characteristics for independently lost CNEs indicating that they have little or no pleiotropy. Length, constraint and ancestry are likely indicators of pleiotropy for the following reasons. First, ancient CNEs that arose in the vertebrate ancestor usually have only a small core of sequence conservation to fish species, while mammals often exhibit much broader conserved flanks (31,45). It was shown that a small conserved core alone is sufficient for transcriptional enhancer activity in transgenic zebrafish, suggesting that in the course of mammalian evolution other functions were added to the originally smaller core enhancer (45). Therefore, an increased CNE length is consistent with added functionality and thus pleiotropy. Second, stronger constraint was observed for genomic regions that encode more than one function (46). Examples include coding exons having a dual role as transcriptional enhancers (47–49) and coding exons that encode information for conserved alternative splice events (50,51). Third, evolution often tinkers with present elements, co-opting both genes (52) and regulatory elements (16) into new functional roles (53). Thus, the older a functional element is, the more likely it is that novel functions were added to existing elements, which might explain our observation that pleiotropic enhancers and CNEs with no losses are evolutionarily older.

Future sequencing efforts (54) and advances in sequencing technologies will produce many more high-quality genomes in the coming years. This will likely allow identifying many additional independent CNE losses and larger CNE loss sets will increase the power to detect enriched functional annotations. The independent CNE losses identified here (Supplementary Table S9) raise interesting biological questions about the consequence of these CNE losses on gene regulation and organism fitness. While it often requires hard work to find the genomic changes associated to given phenotypic changes in a forward genetics approach, our set of (independent) CNE losses provide candidates that can be directly experimentally explored by enhancer assays or genomic knockouts (similar to a reverse genetics approach) to test what the functional consequences and potential phenotypic changes are. Similar to the independent CNE loss underlying pelvic spine loss in sticklebacks (14), these CNE losses may hold important clues for our understanding of the evolution of mammalian phenotype diversity.

## SUPPLEMENTARY DATA

Supplementary Data are available at NAR Online: Supplementary Tables 1–9, Supplementary Figures 1–13 and Supplementary References [55–58].

## FUNDING

Fellowships from the German Research Foundation [Hi 1423/2-1 to M.H.]; Human Frontier Science Program [LT000896/2009-L to M.H.]; NIH [R01HD059862, R01HG005058 to G.B.]; NSF Center for Science of Information (CSoI) under grant agreement [CCF-0939370 to G.B.]. G.B. is a Packard Fellow and Microsoft Faculty Fellow. Funding for open access charge: Packard Foundation.

*Conflict of interest statement*. None declared.

## Supplementary Material

Supplementary Data

## References

[1] Waterston RH, Lindblad-Toh K, Birney E, Rogers J, Abril JF, Agarwal P, Agarwala R, Ainscough R, Alexandersson M, An P (2002). Initial sequencing and comparative analysis of the mouse genome. Nature.

[2] Siepel A, Bejerano G, Pedersen JS, Hinrichs AS, Hou M, Rosenbloom K, Clawson H, Spieth J, Hillier LW, Richards S (2005). Evolutionarily conserved elements in vertebrate, insect, worm, and yeast genomes. Genome Res..

[3] Mikkelsen TS, Wakefield MJ, Aken B, Amemiya CT, Chang JL, Duke S, Garber M, Gentles AJ, Goodstadt L, Heger A (2007). Genome of the marsupial Monodelphis domestica reveals innovation in non-coding sequences. Nature.

[4] Lindblad-Toh K, Garber M, Zuk O, Lin MF, Parker BJ, Washietl S, Kheradpour P, Ernst J, Jordan G, Mauceli E (2011). A high-resolution map of human evolutionary constraint using 29 mammals. Nature.

[5] Woolfe A, Goodson M, Goode DK, Snell P, McEwen GK, Vavouri T, Smith SF, North P, Callaway H, Kelly K (2005). Highly conserved non-coding sequences are associated with vertebrate development. PLoS Biol..

[6] Birney E, Stamatoyannopoulos JA, Dutta A, Guigo R, Gingeras TR, Margulies EH, Weng Z, Snyder M, Dermitzakis ET, Thurman RE (2007). Identification and analysis of functional elements in 1% of the human genome by the ENCODE pilot project. Nature.

[7] Heintzman ND, Stuart RK, Hon G, Fu Y, Ching CW, Hawkins RD, Barrera LO, Van Calcar S, Qu C, Ching KA (2007). Distinct and predictive chromatin signatures of transcriptional promoters and enhancers in the human genome. Nat. Genet..

[8] Visel A, Prabhakar S, Akiyama JA, Shoukry M, Lewis KD, Holt A, Plajzer-Frick I, Afzal V, Rubin EM, Pennacchio LA (2008). Ultraconservation identifies a small subset of extremely constrained developmental enhancers. Nat. Genet..

[9] Ragvin A, Moro E, Fredman D, Navratilova P, Drivenes O, Engstrom PG, Alonso ME, de la Calle Mustienes E, Gomez Skarmeta JL, Tavares MJ (2010). Long-range gene regulation links genomic type 2 diabetes and obesity risk regions to HHEX, SOX4, and IRX3. Proc. Natl Acad. Sci. USA.

[10] Wasserman NF, Aneas I, Nobrega MA (2010). An 8q24 gene desert variant associated with prostate cancer risk confers differential in vivo activity to a MYC enhancer. Genome Res..

[11] Visel A, Rubin EM, Pennacchio LA (2009). Genomic views of distant-acting enhancers. Nature.

[12] McLean C, Bejerano G (2008). Dispensability of mammalian DNA. Genome Res..

[13] Bejerano G, Pheasant M, Makunin I, Stephen S, Kent WJ, Mattick JS, Haussler D (2004). Ultraconserved elements in the human genome. Science.

[14] Chan YF, Marks ME, Jones FC, Villarreal G, Shapiro MD, Brady SD, Southwick AM, Absher DM, Grimwood J, Schmutz J (2010). Adaptive evolution of pelvic reduction in sticklebacks by recurrent deletion of a Pitx1 enhancer. Science.

[15] McLean CY, Reno PL, Pollen AA, Bassan AI, Capellini TD, Guenther C, Indjeian VB, Lim X, Menke DB, Schaar BT (2011). Human-specific loss of regulatory DNA and the evolution of human-specific traits. Nature.

[16] Prud'homme B, Gompel N, Rokas A, Kassner VA, Williams TM, Yeh SD, True JR, Carroll SB (2006). Repeated morphological evolution through cis-regulatory changes in a pleiotropic gene. Nature.

[17] Frankel N, Davis GK, Vargas D, Wang S, Payre F, Stern DL (2010). Phenotypic robustness conferred by apparently redundant transcriptional enhancers. Nature.

[18] Hoekstra HE, Coyne JA (2007). The locus of evolution: evo devo and the genetics of adaptation. Evolution.

[19] Carroll SB (2008). Evo-devo and an expanding evolutionary synthesis: a genetic theory of morphological evolution. Cell.

[20] Carroll SB (2005). Evolution at two levels: on genes and form. PLoS Biol..

[21] Wray GA (2007). The evolutionary significance of cis-regulatory mutations. Nat. Rev. Genet..

[22] Jones FC, Grabherr MG, Chan YF, Russell P, Mauceli E, Johnson J, Swofford R, Pirun M, Zody MC, White S (2012). The genomic basis of adaptive evolution in threespine sticklebacks. Nature.

[23] Dreszer TR, Karolchik D, Zweig AS, Hinrichs AS, Raney BJ, Kuhn RM, Meyer LR, Wong M, Sloan CA, Rosenbloom KR (2012). The UCSC Genome Browser database: extensions and updates 2011. Nucleic Acids Res..

[24] Schwartz S, Kent WJ, Smit A, Zhang Z, Baertsch R, Hardison RC, Haussler D, Miller W (2003). Human-mouse alignments with BLASTZ. Genome Res..

[25] Kent WJ, Baertsch R, Hinrichs A, Miller W, Haussler D (2003). Evolution's cauldron: duplication, deletion, and rearrangement in the mouse and human genomes. Proc. Natl Acad. Sci. USA.

[26] Blanchette M, Kent WJ, Riemer C, Elnitski L, Smit AF, Roskin KM, Baertsch R, Rosenbloom K, Clawson H, Green ED (2004). Aligning multiple genomic sequences with the threaded blockset aligner. Genome Res..

[27] Cooper GM, Stone EA, Asimenos G, Green ED, Batzoglou S, Sidow A (2005). Distribution and intensity of constraint in mammalian genomic sequence. Genome Res..

[28] Pollard KS, Hubisz MJ, Rosenbloom KR, Siepel A (2010). Detection of nonneutral substitution rates on mammalian phylogenies. Genome Res..

[29] Kent WJ (2002). BLAT–the BLAST-like alignment tool. Genome Res..

[30] Flicek P, Amode MR, Barrell D, Beal K, Brent S, Chen Y, Clapham P, Coates G, Fairley S, Fitzgerald S (2011). Ensembl 2011. Nucleic Acids Res..

[31] Prabhakar S, Poulin F, Shoukry M, Afzal V, Rubin EM, Couronne O, Pennacchio LA (2006). Close sequence comparisons are sufficient to identify human cis-regulatory elements. Genome Res..

[32] Blow MJ, McCulley DJ, Li Z, Zhang T, Akiyama JA, Holt A, Plajzer-Frick I, Shoukry M, Wright C, Chen F (2010). ChIP-Seq identification of weakly conserved heart enhancers. Nat. Genet..

[33] DiLeone RJ, Russell LB, Kingsley DM (1998). An extensive 3′ regulatory region controls expression of Bmp5 in specific anatomical structures of the mouse embryo. Genetics.

[34] McPherron AC, Lawler AM, Lee SJ (1999). Regulation of anterior/posterior patterning of the axial skeleton by growth/differentiation factor 11. Nat. Genet..

[35] Liu JP (2006). The function of growth/differentiation factor 11 (Gdf11) in rostrocaudal patterning of the developing spinal cord. Development.

[36] Shi Y, Liu JP (2011). Gdf11 facilitates temporal progression of neurogenesis in the developing spinal cord. J. Neurosci., Off. J. Soc. Neurosci..

[37] Bione S, Sala C, Manzini C, Arrigo G, Zuffardi O, Banfi S, Borsani G, Jonveaux P, Philippe C, Zuccotti M (1998). A human homologue of the Drosophila melanogaster diaphanous gene is disrupted in a patient with premature ovarian failure: evidence for conserved function in oogenesis and implications for human sterility. Am. J. Hum. Genet..

[38] McLean CY, Bristor D, Hiller M, Clarke SL, Schaar BT, Lowe CB, Wenger AM, Bejerano G (2010). GREAT improves functional interpretation of cis-regulatory regions. Nat. Biotechnol..

[39] Visel A, Blow MJ, Li Z, Zhang T, Akiyama JA, Holt A, Plajzer-Frick I, Shoukry M, Wright C, Chen F (2009). ChIP-seq accurately predicts tissue-specific activity of enhancers. Nature.

[40] Green RE, Krause J, Briggs AW, Maricic T, Stenzel U, Kircher M, Patterson N, Li H, Zhai W, Fritz MH (2010). A draft sequence of the Neandertal genome. Science.

[41] Kalay G, Wittkopp PJ (2010). Nomadic enhancers: tissue-specific cis-regulatory elements of yellow have divergent genomic positions among Drosophila species. PLoS Genet..

[42] Arendt J, Reznick D (2008). Convergence and parallelism reconsidered: what have we learned about the genetics of adaptation?. Trends Ecol. Evol..

[43] Gompel N, Prud'homme B (2009). The causes of repeated genetic evolution. Dev. Biol..

[44] Stern DL, Orgogozo V (2009). Is genetic evolution predictable?. Science.

[45] McEwen GK, Goode DK, Parker HJ, Woolfe A, Callaway H, Elgar G (2009). Early evolution of conserved regulatory sequences associated with development in vertebrates. PLoS Genet..

[46] Lin MF, Kheradpour P, Washietl S, Parker BJ, Pedersen JS, Kellis M (2011). Locating protein-coding sequences under selection for additional, overlapping functions in 29 mammalian genomes. Genome Res..

[47] Dong X, Navratilova P, Fredman D, Drivenes O, Becker TS, Lenhard B (2009). Exonic remnants of whole-genome duplication reveal cis-regulatory function of coding exons. Nucleic Acids Res..

[48] Lampe X, Samad OA, Guiguen A, Matis C, Remacle S, Picard JJ, Rijli FM, Rezsohazy R (2008). An ultraconserved Hox-Pbx responsive element resides in the coding sequence of Hoxa2 and is active in rhombomere 4. Nucleic Acids Res..

[49] Birnbaum RY, Clowney EJ, Agamy O, Kim MJ, Zhao J, Yamanaka T, Pappalardo Z, Clarke SL, Wenger AM, Nguyen L (2012). Coding exons function as tissue-specific enhancers of nearby genes. Genome Res..

[50] Sorek R, Shemesh R, Cohen Y, Basechess O, Ast G, Shamir R (2004). A non-EST-based method for exon-skipping prediction. Genome Res..

[51] Sugnet CW, Kent WJ, Ares M, Haussler D (2004). Transcriptome and genome conservation of alternative splicing events in humans and mice. Pac. Symp. Biocomput..

[52] Jeffery CJ (2009). Moonlighting proteins–an update. Mol. Biosyst..

[53] Prud'homme B, Gompel N, Carroll SB (2007). Emerging principles of regulatory evolution. Proc. Natl Acad. Sci. USA.

[54] Haussler D, O'Brien S, Ryder O, Barker F, Clamp M, Crawford A, Hanner R, Hanotte O, Johnson W, McGuire J (2009). Genome 10K: a proposal to obtain whole-genome sequence for 10,000 vertebrate species. J. Hered..

[55] Hedges SB, Dudley J, Kumar S (2006). TimeTree: a public knowledge-base of divergence times among organisms. Bioinformatics.

[56] Visel A, Minovitsky S, Dubchak I, Pennacchio LA (2007). VISTA Enhancer Browser–a database of tissue-specific human enhancers. Nucleic Acids Res..

[57] Persampieri J, Ritter DI, Lees D, Lehoczky J, Li Q, Guo S, Chuang JH (2008). cneViewer: a database of conserved non-coding elements for studies of tissue-specific gene regulation. Bioinformatics.

[58] Abd El-Aziz MM, Barragan I, O'Driscoll CA, Goodstadt L, Prigmore E, Borrego S, Mena M, Pieras JI, El-Ashry MF, Safieh LA (2008). EYS, encoding an ortholog of Drosophila spacemaker, is mutated in autosomal recessive retinitis pigmentosa. Nat. Genet..

