# Supplementary material for: Hundreds of conserved non-coding genomic regions are independently lost in mammals
Source: Nucleic Acids Res. 2012 Oct 5;40(22):11463–76. doi: 10.1093/nar/gks905 (PMC3526296; doi:10.1093/nar/gks905)
Supplement: Supplementary Data [file supp_40_22_11463__index.html]

Hundreds of conserved non-coding genomic regions are independently lost in mammals — Hundreds of conserved non-coding genomic regions are independently lost in mammals — Supplementary Data 

# Hundreds of conserved non-coding genomic regions are independently lost in mammals

## Supplementary Data

files

**Files in this Data Supplement:**

- Supplementary Data - docx file
- Supplementary Data - xls file
