# Supplementary material for: Hundreds of conserved non-coding genomic regions are independently lost in mammals
Source: Nucleic Acids Res. 2012 Oct 5;40(22):11463–76. doi: 10.1093/nar/gks905 (PMC3526296; doi:10.1093/nar/gks905)
Supplement: Supplementary Data [file supp_gks905_nar-01139-x-2012-File008.docx]

**Supplementary Material for**

Hundreds of conserved non-coding genomic regions

are independently lost in mammals

Michael Hiller ^1*^, Bruce T. Schaar ^1^ and Gill Bejerano ^1,2^

^1^ Department of Developmental Biology, Stanford University, Stanford, California 94305, USA

^2^ Department of Computer Science, Stanford University, Stanford, California 94305, USA

^*^ present address: Max Planck Institute of Molecular Cell Biology and Genetics & Max Planck Institute for the Physics of Complex Systems, Dresden, Germany

Correspondence should be addressed to M.H. [hiller@mpi-cbg.de](mailto:hiller@mpi-cbg.de) and G.B. [bejerano@stanford.edu](mailto:bejerano@stanford.edu)

The Supplementary material contains

- Supplementary Figures 1 to 13
- Supplementary Tables 1 to 9
- Supplementary References 55-58

Table 9 containing the three sets of independently lost CNEs is provided as a separate Excel file.


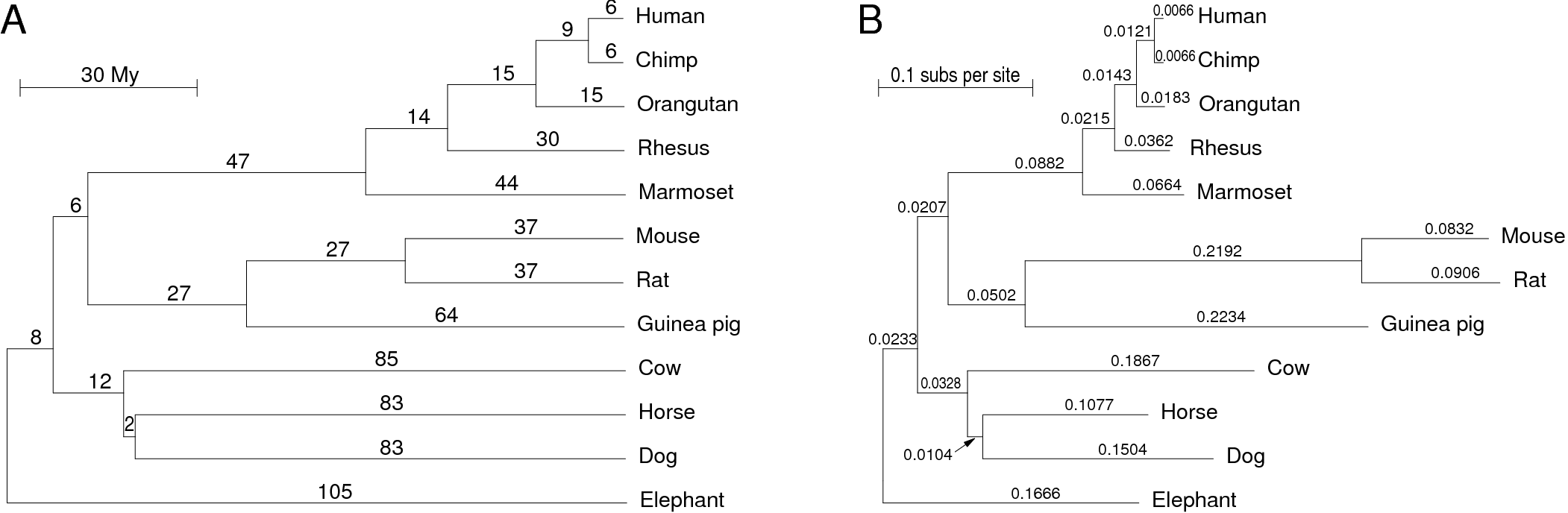


Supplementary Figure 1: Phylogenetic tree of the species where we search for CNE losses.

(A) Scaled by divergence times in million years (My) taken from [www.timetree.org](http://www.timetree.org) ([55](#_ENREF_55)) (timetree expert result or if that is not available weighted average of all studies).

(B) Scaled by branch length taken from

http://hgdownload.cse.ucsc.edu/goldenPath/hg18/phastCons44way/vertebrate.mod


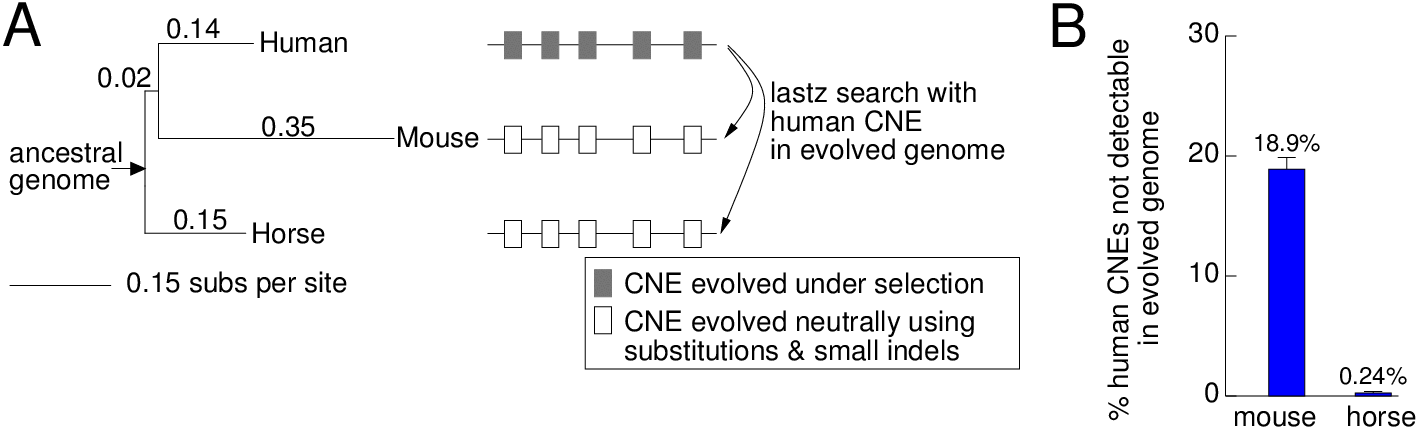


Supplementary Figure 2: Most CNE losses likely involve large insertions/deletions (indels).

(A) By simulating genome evolution, we tested whether substitutions and small indels alone are sufficient to change the CNE sequence to an extent that no sequence similarity can be detected. To this end, we evolved an ancestral genome along this phylogeny, letting CNEs in mouse and horse evolve neutrally, allowing only substitutions and small indels to occur (large insertions and deletions did not occur). Then we used lastz to find the CNE (sequence taken from the evolved human genome) in the evolved mouse/horse genome. We selected mouse and horse as these species have the longest and shortest branch length, respectively, among the species where we search for CNE losses.

(B) Only 0.2% of the CNEs are not detectable in the CNE-loss genome for the short branch length (horse). 18.9% of the CNEs are not detectable for the longer branch length (mouse). This suggests that – while some of our complete CNE losses can be due to the accumulation of many small events – most CNE losses involve large indels, which is in agreement with the results in Fig. 1D. Note that this represents an upper bound as evolving mouse for 0.35 and horse for 0.15 substitutions per site represents CNE loss directly after the split of these lineages. A more recent CNE loss would result in a lower number of neutral mutations and therefore a higher chance of being detected by lastz. Error bars are standard deviation over five independent evolution simulations.


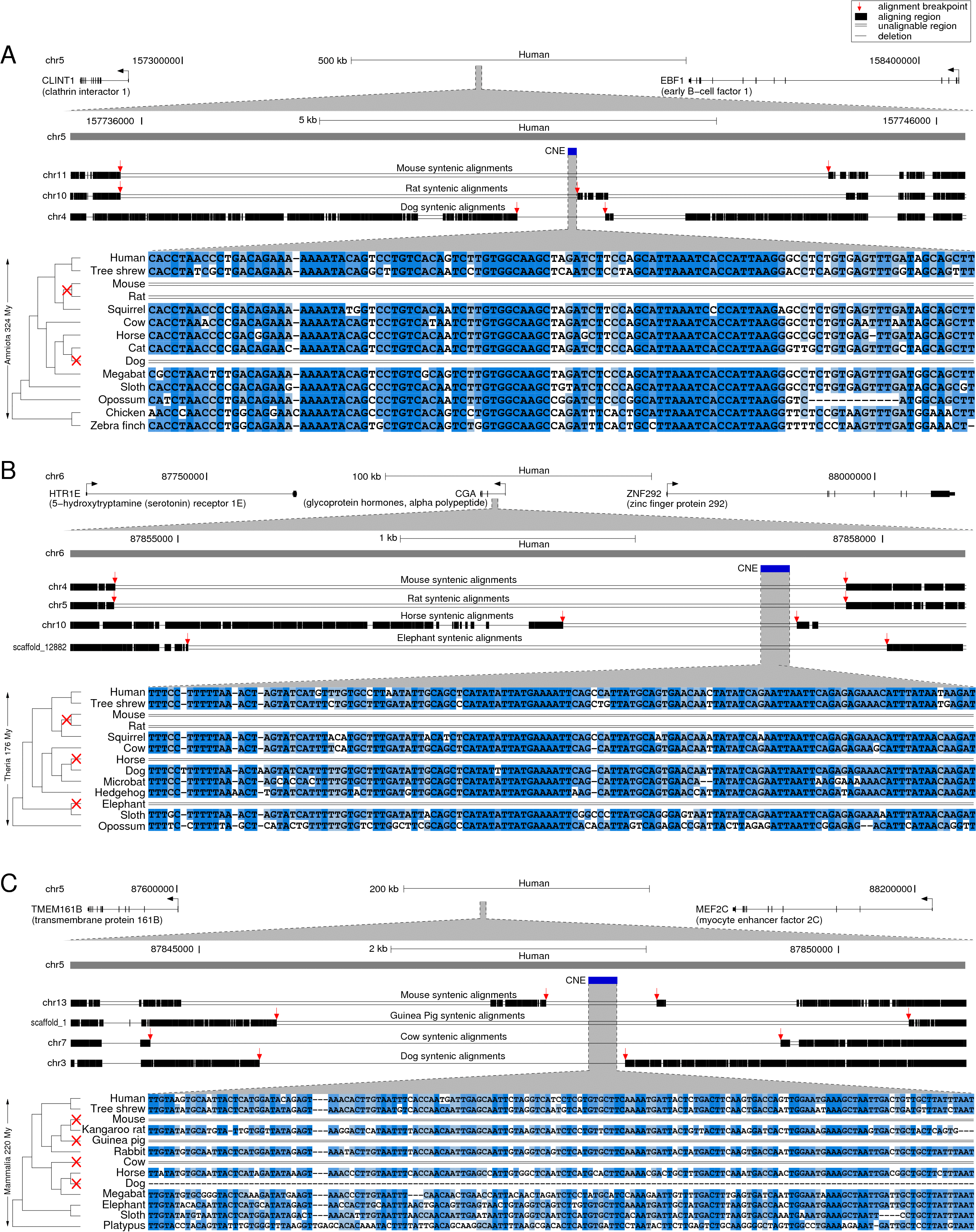


Supplementary Figure 3: Examples of independently lost CNEs.

(A) Two independent losses in the mouse-rat ancestor and in the dog lineage. (B) Three independent losses in the mouse-rat ancestor, in the horse and in the elephant lineage. (C) Four independent losses in the mouse, guinea pig, cow and dog lineage. Legend as in Fig. 2A main text.


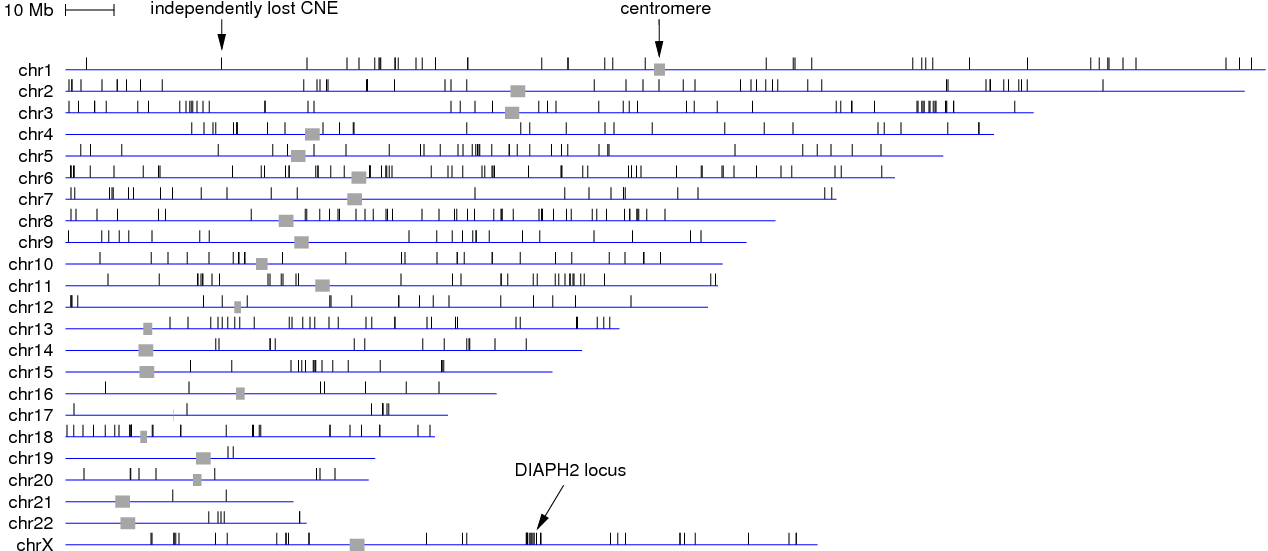


Supplementary Figure 4: Position in the human genome of the 619 CNEs with independent losses in mammals.

The *DIAPH2* locus that is displayed in detail in Figure 3 main text is indicated. Centromeres are shown as grey boxes.


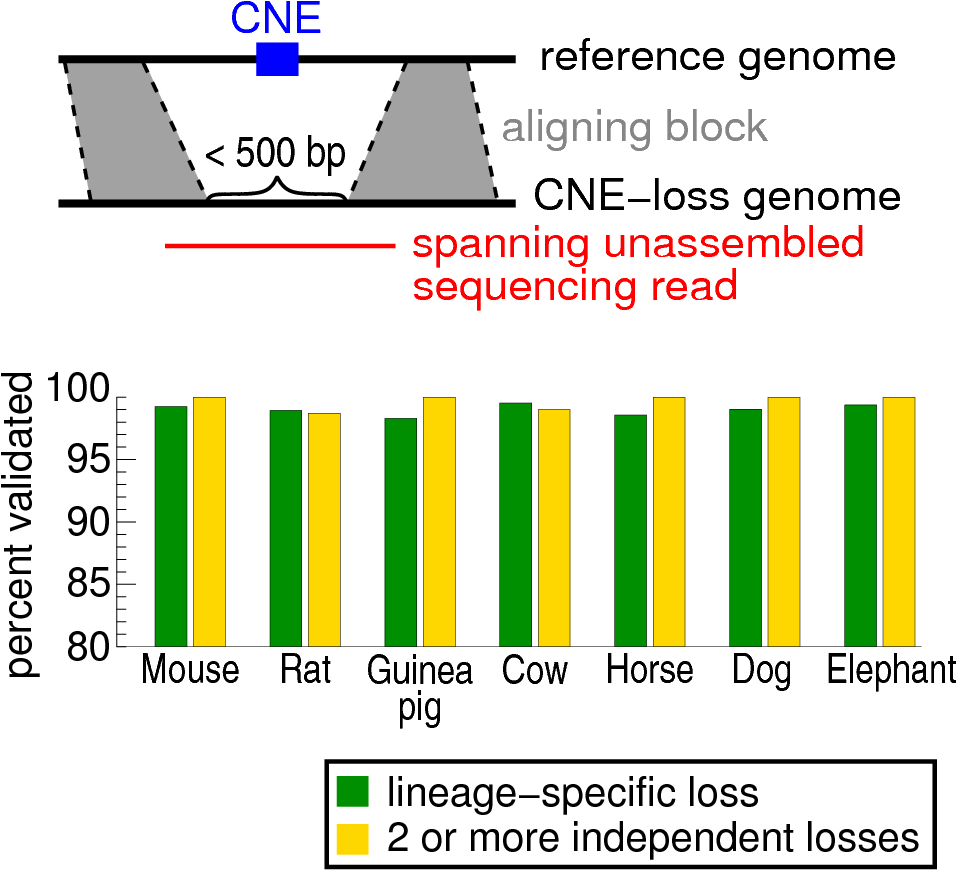


Supplementary Figure 5: Lineage-specific and independently lost CNEs have similar assembly validation rates.

We validated assembly regions less than 500 bp that comprise a lineage-specific or independent CNE loss by single unassembled sequencing reads that span the upstream and downstream aligning block.


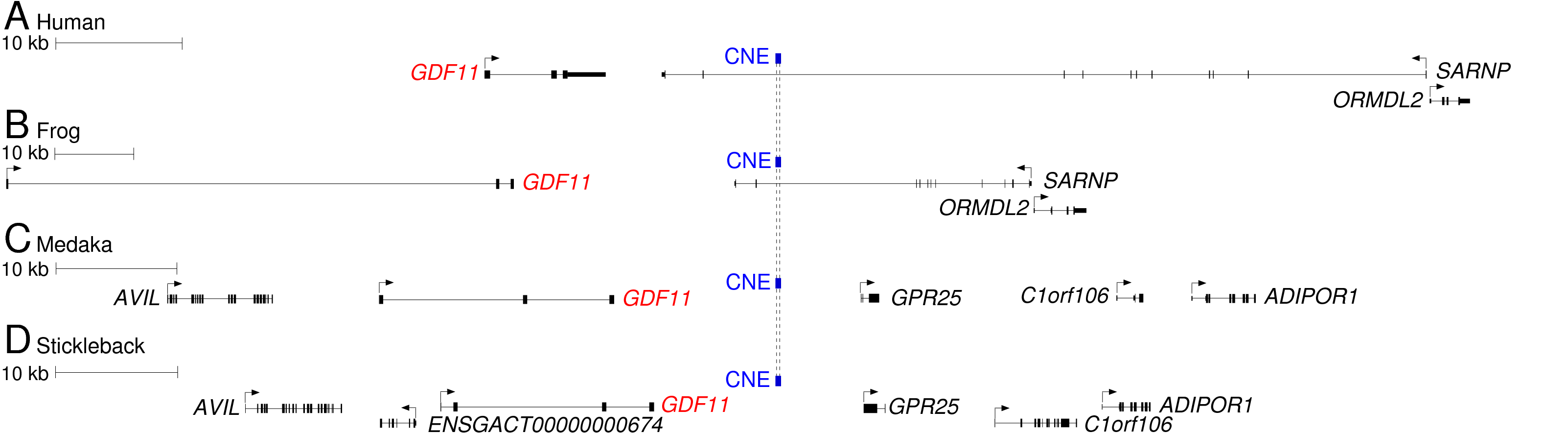


Supplementary Figure 6: Conserved synteny between the tested CNE and *GDF11*.

The CNE that encodes a spinal cord enhancer (Fig. 2) is in synteny with *GDF11* in human (A), frog (B), medaka (C) and stickleback (D). Synteny to *SARNP* and *ORMDL2* is only found in tetrapods but not fish.


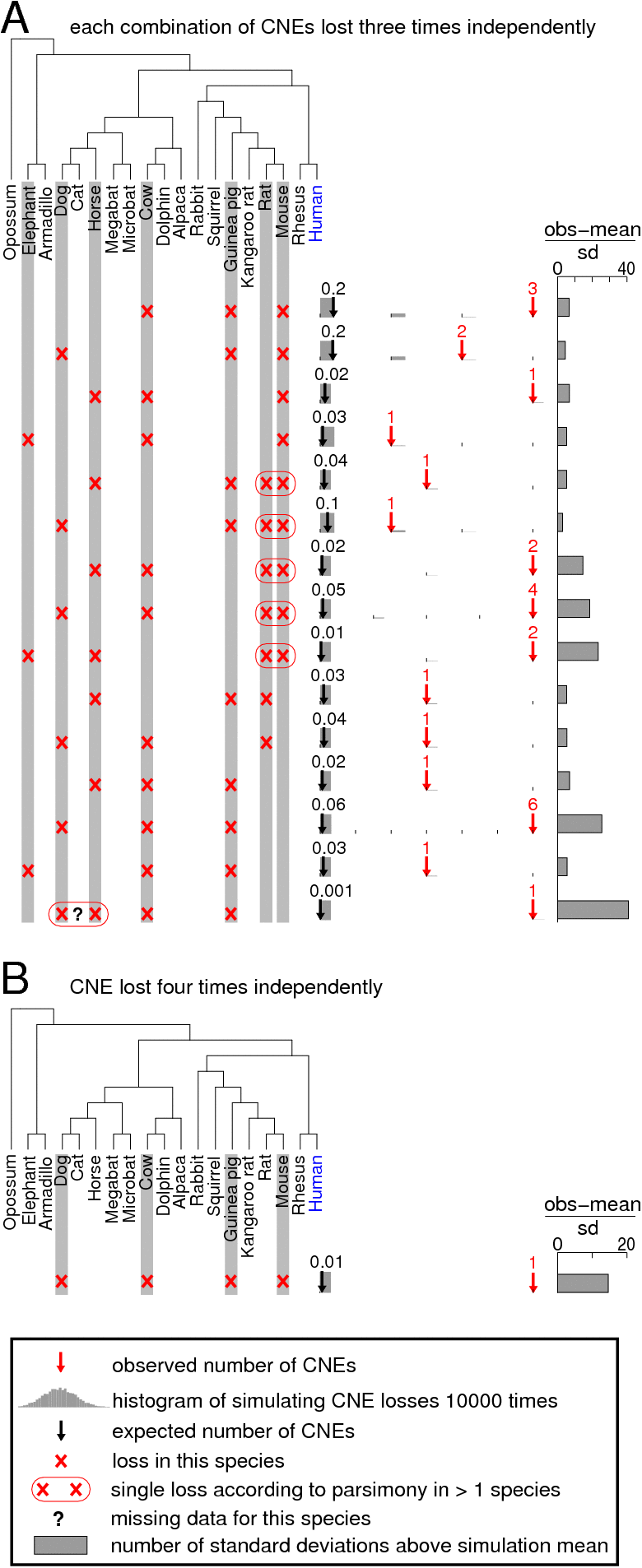


Supplementary Figure 7:

All observed combinations of three (A) and four (B) independent CNE losses for the data set where human is the reference genome. Legend as in Figure 4 main text.


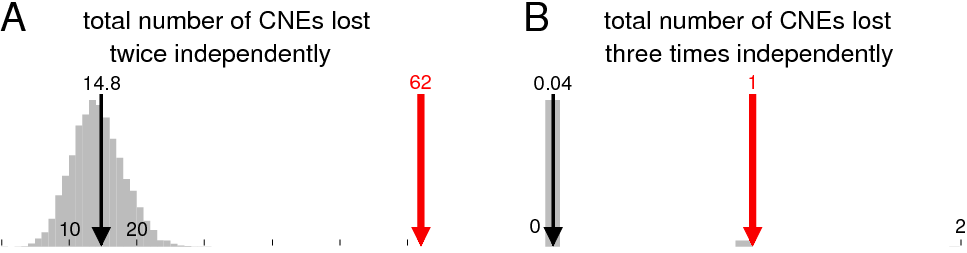


Supplementary Figure 8: Independent CNE losses for the subset of CNEs that is highly constrained.

Under uniform loss frequencies, we expect 14.8 CNEs lost twice (A) and 0.04 CNEs lost three-times (B), which is significantly less than observed (empirical P-value < 0.0001, z-score = 12.4 for CNEs lost twice; P-value = 0.04, z-score = 4.7 for the CNE lost three-times). Human is the reference genome. Legend as in Figure 4 main text.


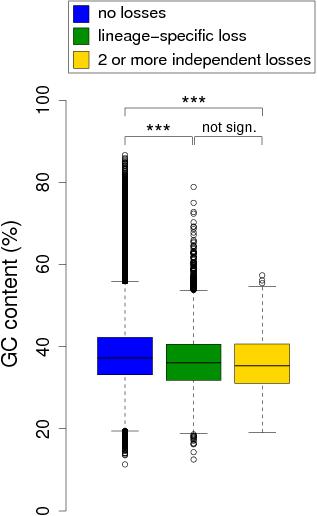


Supplementary Figure 9: GC content for human sequences of CNEs with no detected losses, lineage-specific or independent losses shows significant differences between the three groups, however the absolute differences are small and it is unclear if they are biologically meaningful (38.14%, 36.78%, 36.18% GC for CNEs with no detected, lineage-specific and independent losses). Human was used as the reference species. *** P-value < 0.0001

Supplementary Figure 10: Dinucleotide frequencies for human sequences of CNEs with no, lineage-specific or independent losses show only slight differences between the three groups. Human was used as the reference species.


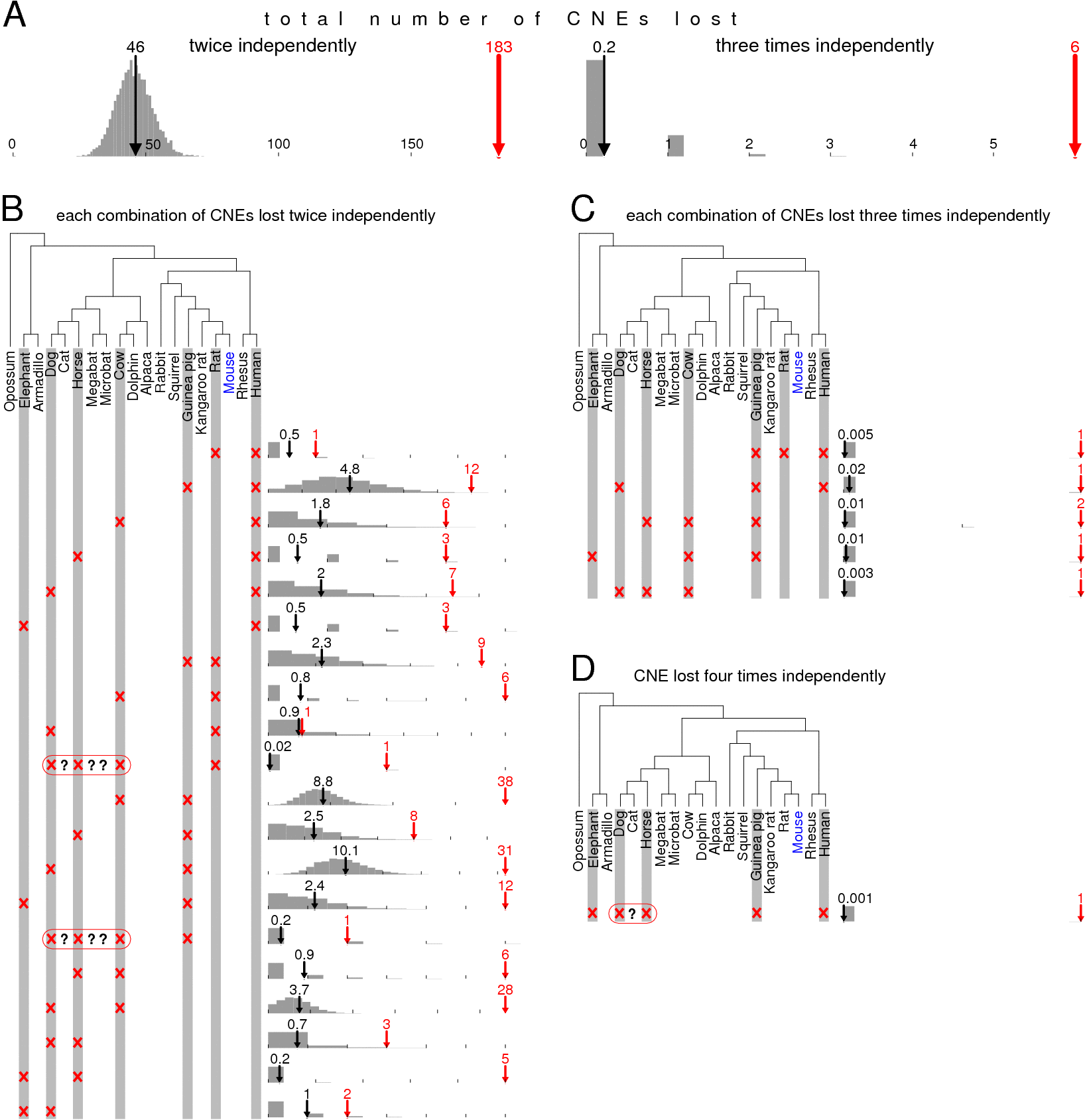


Supplementary Figure 11: Independent CNE losses using mouse as the reference genome.

(A) We observed a total of 183 CNEs lost twice, six lost three-times, which is significantly more than expected under uniform CNE loss frequencies (P-values < 0.0001, <0.0001 and z-scores 20.5, 12.5 for two and three independent losses, respectively).

(B-D) All observed combinations of two (B), three (C) and four (D) independent CNE losses.

Legend as in Figure 4 main text.


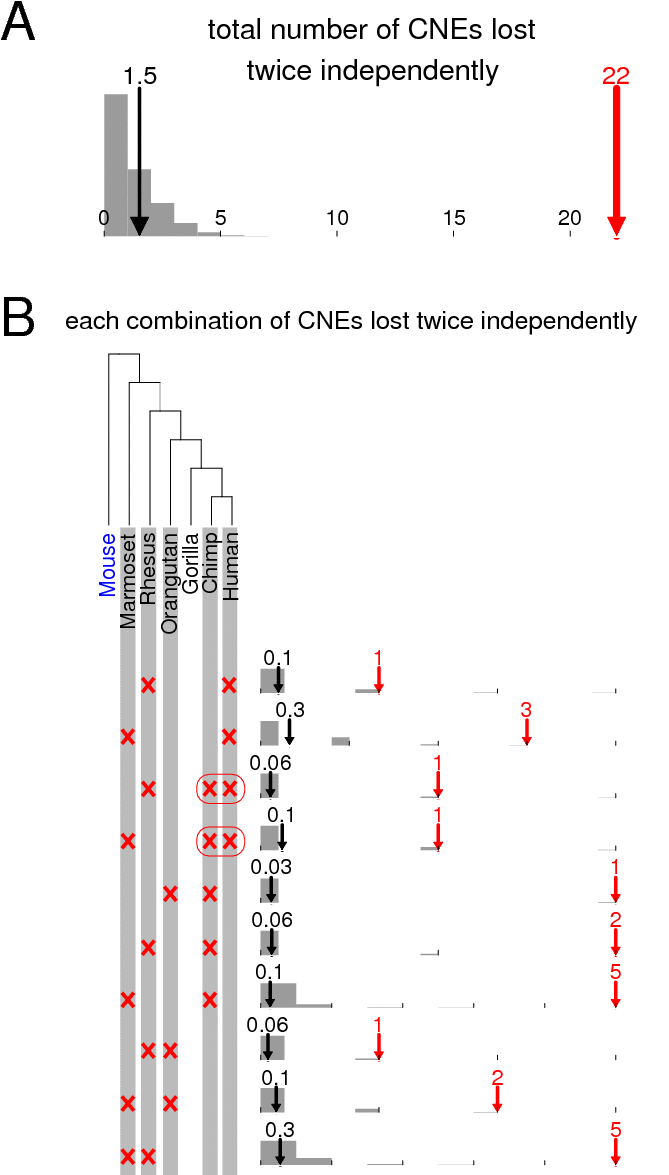


Supplementary Figure 12: Independent CNE losses in the primate clade.

(A) Total number of two independent CNE losses. Under uniform loss frequencies, we expect 1.5 CNEs lost twice, which is significantly less than the 22 observed losses (empirical P-value < 0.0001, z-score = 16.6).

(B) All combinations of two independent CNE losses.

Mouse was used as the reference species. Legend as in Figure 4 main text.


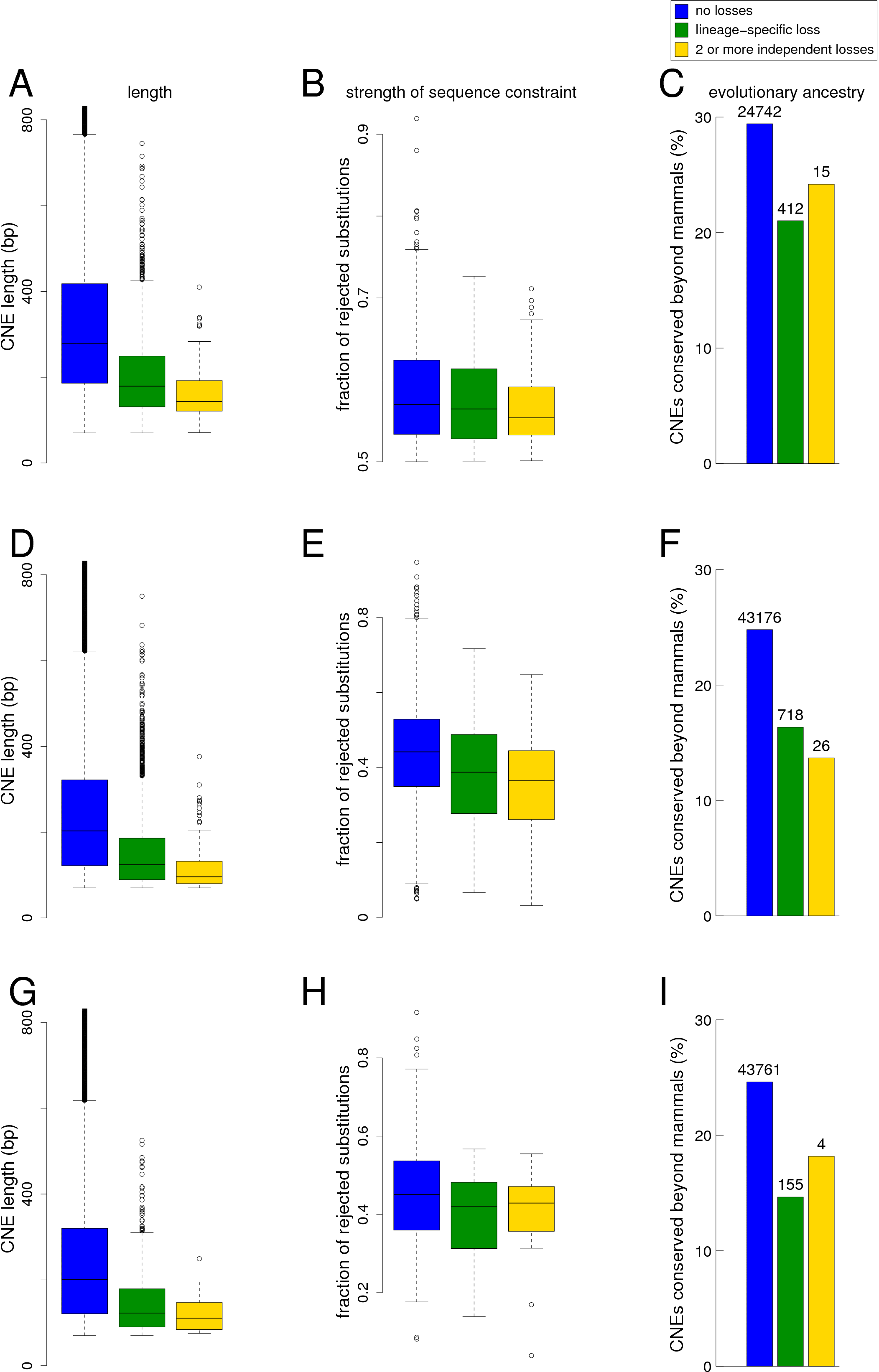


Supplementary Figure 13: Length, sequence constraint and evolutionary ancestry of CNEs with no losses (blue), lineage-specific losses (green) and two or more independent losses (yellow).

(A-C) Human is the reference species, highly constrained subset. (D-F) Mouse is the reference species. (G-I) Mouse is the reference species, losses in the primate lineage only. Consistently for these data sets, we found that CNE length decreases with the number of loss events (A,D,G). CNEs with loss events are depleted in extremely conserved elements (B,E,H). CNEs with loss events align less frequently to non-mammalian vertebrates (chicken, zebra finch, lizard, frog or fish species), indicating a more recent evolutionary ancestry (C,F,I). For visualization clarity the Y-axis in (A,D,G) is cut a size of 800 bp. Size-matched CNEs are compared in (B,E,H) to remove the effect of the confounding variable ‘length’.

|  | Total number | Number of less pleiotropic enhancers |
| --- | --- | --- |
| VISTA enhancers | 721 | 406 (56%) |
| CNEViewer enhancers | 72 | 31 (43%) |

Supplementary Table 1: Pleiotropy of tested enhancers.

We analyzed how many enhancers tested in mouse (VISTA enhancer database ([56](#_ENREF_56))) and how many enhancers tested in zebrafish (CNEViewer database ([57](#_ENREF_57))) drive expression in only one tissue (less pleiotropic). As we cannot exclude that an enhancer drives expression in a different tissue at another timepoint, we use less pleiotropic instead of non-pleiotropic. CNEViewer data was obtained from <http://bioinformatics.bc.edu/chuanglab/cneBrowser/zebramouse2.php>. We extracted positively tested enhancers and counted how many enhancers have more than one expression domain, excluding non-specific patterns. We downloaded Vista enhancers from [http://enhancer.lbl.gov](http://enhancer.lbl.gov/), extracted the positively tested enhancers and counted the number of listed expression domains per enhancer.

Supplementary Table 2: Clusters of independently lost CNEs.

The table lists the locus of sliding windows containing several independently lost CNEs and the RefSeq genes located in the window. The window around the *DIAPH2* gene that contains the highest number of independently lost CNEs is shown in Fig. 3 main text.

Supplementary Table 3: Number of observed, expected and simulated independent CNE losses and number of CNE losses affected by overlapping large-scale events. The table is sorted by number of losses observed over the normalized simulation average. Losses in the ancestor of two or more species are indicated by a hyphen and have losses or missing data for all descendant species (e.g. cow-horse is a single loss in the ancestor of cow and horse (inferred by parsimony)). Human was used as the reference species.

|  |
| --- |

Supplementary Table 4: Number of observed, expected and simulated independent CNE losses for the subset of highly conserved elements. The table is sorted by number of losses observed over the normalized simulation average. Losses in the ancestor of two or more species are indicated by a hyphen and have losses or missing data for all descendant species. Human was used as the reference species.

|  | | |  |  |  |
| --- | --- | --- | --- | --- | --- |
| chr | start | end | CNE-loss species | nearest gene | comments |
| **gene loss in subset of CNE-loss species** | | | | | |
| chr8 | 118111991 | 118112147 | mouse,rat, guinea pig | *SLC30A8* | gene loss in guinea pig |
| chr1 | 239134987 | 239135112 | mouse,rat, cow | *GREM2* | gene loss in cow |
| chr18 | 64758640 | 64758806 | mouse, horse | *CCDC102B* | gene loss in mouse |
| chr18 | 64763738 | 64763944 | mouse, horse | *CCDC102B* | gene loss in mouse |
| chr4 | 111171254 | 111171412 | guinea pig, cow | *EGF* | gene loss in cow |
| chr6 | 67270443 | 67270548 | rat, dog | *EYS* | gene loss in rat |
| **gene loss in all CNE-loss species** | | | | | |
| chr8 | 52387808 | 52387896 | guinea pig, cow | *PXDNL* |  |
| chr6 | 65121465 | 65121541 | mouse, guinea pig | *EYS* |  |
| chr6 | 65956300 | 65956420 | cow, guinea pig | *EYS* |  |
| chr6 | 66168383 | 66168595 | mouse, guinea pig | *EYS* |  |
| chr6 | 66691546 | 66691761 | cow, guinea pig | *EYS* |  |

Supplementary Table 5: CNE losses that are associated with gene losses.

Coordinates are for the human hg18 assembly. *EYS* loss in mouse, rat, guinea pig and cow is described in ([58](#_ENREF_58)). *EYS* lacks inactivating mutations in dog. The loss of the other genes has not been described in the literature to the best of our knowledge.

Supplementary Table 6: Number of observed, expected and simulated independent CNE losses using mouse as the reference species. The table is sorted by number of losses observed over the normalized simulation average. Losses in the ancestor of two or more species are indicated by a hyphen and have losses or missing data for all descendant species.

Species assembly coverage unassembled Sanger sequencing data (Gb)

human complete 204

chimp 6X 43

orangutan 6X 32

rhesus 5.1X 30

marmoset 6X 25

Supplementary Table 7: Assembly coverage and publicly available sequence data in unassembled traces for the primate species where we search for CNE losses.

Supplementary Table 8: Number of observed, expected and simulated independent CNE losses in the primate lineage only using mouse as the reference species. The table is sorted by number of losses observed over the normalized simulation average. Losses in the ancestor of two or more species are indicated by a hyphen and have losses or missing data for all descendant species.

**Supplementary References**

55. Hedges, S.B., Dudley, J. and Kumar, S. (2006) TimeTree: a public knowledge-base of divergence times among organisms. *Bioinformatics*, **22**, 2971-2972.

56. Visel, A., Minovitsky, S., Dubchak, I. and Pennacchio, L.A. (2007) VISTA Enhancer Browser--a database of tissue-specific human enhancers. *Nucleic Acids Res*, **35**, D88-92.

57. Persampieri, J., Ritter, D.I., Lees, D., Lehoczky, J., Li, Q., Guo, S. and Chuang, J.H. (2008) cneViewer: a database of conserved non-coding elements for studies of tissue-specific gene regulation. *Bioinformatics*, **24**, 2418-2419.

58. Abd El-Aziz, M.M., Barragan, I., O'Driscoll, C.A., Goodstadt, L., Prigmore, E., Borrego, S., Mena, M., Pieras, J.I., El-Ashry, M.F., Safieh, L.A. *et al.* (2008) EYS, encoding an ortholog of Drosophila spacemaker, is mutated in autosomal recessive retinitis pigmentosa. *Nat Genet*, **40**, 1285-1287.
